# Pharmacogenetic—Whole blood and intracellular pharmacokinetic—Pharmacodynamic (PG-PK^2^-PD) relationship of tacrolimus in liver transplant recipients

**DOI:** 10.1371/journal.pone.0230195

**Published:** 2020-03-12

**Authors:** Camille Tron, Jean-Baptiste Woillard, Pauline Houssel-Debry, Véronique David, Caroline Jezequel, Michel Rayar, David Balakirouchenane, Benoit Blanchet, Jean Debord, Antoine Petitcollin, Mickaël Roussel, Marie-Clémence Verdier, Eric Bellissant, Florian Lemaitre

**Affiliations:** 1 Rennes 1 University, Rennes University Hospital, Inserm, EHESP, Irset (Institut de recherche en santé, environnement et travail)—UMR_S 1085, Rennes, France; 2 INSERM, CIC 1414 Clinical Investigation Center, Rennes, France; 3 Department of Pharmacology and Toxicology, Limoges University Hospital, Limoges, France; 4 INSERM, UMR 1248, Limoges, France; 5 Limoges University, Limoges, France; 6 Hepato-Biliary and Digestive Surgery Unit, Rennes University Hospital, Rennes, France; 7 Department of Molecular Genetics and Genomics, Rennes University Hospital, Rennes, France; 8 CNRS, UMR6290, IGDR, Rennes, France; 9 Assistance Publique-Hôpitaux de Paris (AP-HP), Pharmacokinetics and Pharmacochemistry Department, Cochin Hospital, Paris, France; 10 CNRS, UMR8638, Faculty of Pharmacy, Paris Descartes University, PRES Sorbonne Paris Cité, Paris, France; 11 Haematology Laboratory, Rennes University Hospital, Rennes, France; Université Paris Descartes, FRANCE

## Abstract

Tacrolimus (TAC) is the cornerstone of immunosuppressive therapy in liver transplantation. This study aimed at elucidating the interplay between pharmacogenetic determinants of TAC whole blood and intracellular exposures as well as the pharmacokinetic-pharmacodynamic relationship of TAC in both compartments. Complete pharmacokinetic profiles (Predose, and 20 min, 40 min, 1h, 2h, 3h, 4h, 6h, 8h, 12h post drug intake) of twice daily TAC in whole blood and peripheral blood mononuclear cells (PBMC) were collected in 32 liver transplanted patients in the first ten days post transplantation. A non-parametric population pharmacokinetic model was applied to explore TAC pharmacokinetics in blood and PBMC. Concurrently, calcineurin activity was measured in PBMC. Influence of donor and recipient genetic polymorphisms of *ABCB1*, *CYP3A4* and *CYP3A5* on TAC exposure was assessed. Recipient *ABCB1* polymorphisms 1199G>A could influence TAC whole blood and intracellular exposure (p<0.05). No association was found between *CYP3A4* or *CYP3A5* genotypes and TAC whole blood or intracellular concentrations. Finally, intra-PBMC calcineurin activity appeared incompletely inhibited by TAC and less than 50% of patients were expected to achieve intracellular IC_50_ concentration (100 pg/millions of cells) at therapeutic whole blood concentration (i.e.: 4–10 ng/mL). Together, these data suggest that personalized medicine regarding TAC therapy might be optimized by ABCB1 pharmacogenetic biomarkers and by monitoring intracellular concentration whereas the relationship between intracellular TAC exposure and pharmacodynamics biomarkers more specific than calcineurin activity should be further investigated.

## 1. Introduction

Tacrolimus (TAC) is an immunosuppressive drug widely prescribed in solid organ transplant patients. Its effect is mediated through the inhibition of intracellular calcineurin (CaN), a serine-threonine phosphatase enzyme, which results in the inhibition of interleukine-2 (IL-2) synthesis by T-lymphocytes [1].

TAC pharmacological response exhibits substantial inter-individual variability. This variability can be partially managed by performing therapeutic drug monitoring (TDM) of TAC whole blood concentrations [2]. Indeed, TDM of TAC is mandatory since it was evidenced that lower whole blood concentrations increase risk of acute rejection (ACR) and that some toxicity are induced by high whole blood concentrations [3].

Despite this personalized approach, some patients experience ACR or toxicity while having blood concentration within the therapeutic range [2]. These observations emphasize the need to look for alternative biomarkers of TAC response.

Measuring TAC directly into its site of effect (i.e. the lymphocyte or, for more practical reason, peripheral blood mononuclear cells (PBMC) a fraction that is enriched in lymphocytes) appears to be a promising strategy to refine TAC TDM. The proof of concept of the clinical interest of TAC measurement in PBMC was established in liver transplant recipients by Capron *et al*. [4]. The authors reported a good correlation between intrahepatic or intra-PBMC concentrations of TAC, and an histological rejection score determined at day-7 post-transplantation whereas no association was found with whole blood TAC concentrations. Besides, the relationship between TAC whole blood concentrations and TAC concentrations in PBMC have been studied in various organ transplant patients [5–11]. These works were concordant to report a poor correlation between trough whole blood and trough intracellular concentrations, meaning that monitoring TAC in its target cell could be a better surrogate marker of its pharmacological effect.

The study of TAC pharmacokinetics in PBMC may be of interest, but it is worthwhile to investigate factors determining drug intracellular disposition. TAC is known to be a substrate of membrane transporters, in particular the efflux transporter ABCB1 (P-glycoprotein or P-gp) expressed in lymphocytes [3,12]. Thus, genetic factors that alter P-gp activity could influence TAC disposition into its target cell. Many single nucleotide polymorphisms (SNP) of *ABCB1* gene (coding for P-gp) have been described. The most extensively studied regarding TAC pharmacokinetics are 1236C>T (rs1128503), 2677G>T/A (rs2032582), and 3435C>T (rs1045642) which are in strong linkage disequilibrium. The influence of these SNPs on TAC whole blood concentrations has been widely investigated and led to conflicting results [13–17]. Nevertheless, some data suggest that *ABCB1* genotype could impact TAC intracellular pharmacokinetics rather than whole blood pharmacokinetics [4,18–20]. In addition, the SNP 1199G>A (rs2229109) was associated with TAC intra-PBMC or intra-hepatic concentrations [18,20,21], and the variant 1199A was associated with an increased risk of kidney allograft loss [22]. Data regarding this SNP and TAC pharmacokinetics are sparse and should be further explored. Besides, CYP3A5 is the major metabolism enzymes of TAC. To date, *CYP3A5* polymorphism is the only genetic marker used in clinical practice because of a clear relationship between *CYP3A5* expression and TAC dose requirement to reach whole blood therapeutic range [23]. In addition, *CYP3A4* genotype could influence TAC pharmacokinetics in particular in *CYP3A5* non-expresser [23]. Nevertheless, it remains to be elucidated whether the most relevant SNP in *CYP3A4* and *CYP3A5* could impact TAC intracellular concentration in comparison to whole blood.

Finally, drug disposition in PBMC could determine the level of inhibition of CaN, the target enzyme. CaN activity in PBMC is intuitively an interesting pharmacodynamic biomarker of the immunosuppressive effect since an increase of CaN activity was suggested to occur before acute cellular rejection [24–26]. A few studies, conducted in liver transplantation, described the pharmacokinetic-pharmacodynamic relationship of TAC by studying the link between whole blood concentrations and CaN activity in PBMC [27–29]. However, the complete relationship between TAC whole blood concentration, TAC intra-PBMC concentration and TAC-induced CaN inhibition, have only been reported in a preliminary work by our team [30], and remains to be widely explored and emphasized with the drug pharmacogenetics.

The aims of the present study was then i) to develop a population pharmacokinetic model using a non-parametric modeling approach to describe whole blood and intracellular pharmacokinetics of tacrolimus, ii) to explore the pharmacogenetic-whole blood and intracellular pharmacokinetic-pharmacodynamic (PG-PK-PK-PD or PG-PK^2^-PD) relationships of TAC in liver transplant recipients in the early period post transplantation.

## 2. Material and methods

### 2.1. Study design

#### 2.1.1. Patients

*De novo* Caucasian liver transplant recipients transplanted between November 2015 and September 2017 in Rennes University Hospital were included in the study which is an ancillary study of the CYPTAC’H protocol (“Pharmacogenetic study of tacrolimus in hepatic transplants » Clinical trial number: NCT01388387). The study protocol was approved by the local ethical committee (Rennes University Hospital). Adult patients, treated with TAC and who gave their written consent to participate in the study, were suitable for inclusion. Nevertheless, patient could not be included if its donor of graft was entered in the national register of refusals (all donors were deceased donors). Patients receiving induction treatment (i.e. anti-lymphocyte or anti-interleukine-2) were excluded because of PBMC (lymphocytes) depletion for several days after transplantation.

#### 2.1.2. Immunosuppressive regimen

TAC treatment was started on post-operative day 0 or 1 (either at 8:00 AM or 8:00 PM, depending on the time the surgical procedure was completed). Patients received initially a dose of 0.04 to 0.05 mg/kg per 12 h or a dose of 0.02 to 0.03 mg/kg per 12 h in case of concomitant administration of fluconazole prophylaxis. TAC whole blood concentrations were monitored daily, then three times a week to maintain trough TAC whole-blood concentrations between 4 and 10 ng/mL. From day-1 post-transplantation, patients concomitantly received oral mycophenolate mofetil 1.5 g twice daily and 20 mg of prednisone once daily. They also received a 500 mg methylprednisolone infusion as an induction and one other 500 mg infusion at portal vein clamp removal. Other co-medications were recorded in the case report form to be sure that patients were not concomitantly treated by CYP450 or ABCB1 inducer or inhibitor at the time of the study.

#### 2.1.3. Data collection

For each patient, 5 milliliters of peripheral venous blood were collected in EDTA tubes at 0, 20, 40, 60, 120, 180, 240, 360, 540 and 720 min after the morning oral dose of twice daily TAC, between the seventh and the tenth day of treatment. No dosage modification occurred within 3 days before sampling to measure concentrations as close as possible to steady state. After whole blood concentrations measurement, the remaining blood was used to obtain complete pharmacokinetic profiles of TAC in PBMC and to measure CaN activity in PBMC at each sampling time. Additional biological parameters were prospectively collected on the day of blood sampling such as albumin, blood count and hematocrit.

### 2.2. Isolation of PBMC

Beforehand measuring TAC concentration and CaN activity in PBMC, cells were isolated from whole blood at each sampling time by density gradient centrifugation according to the procedure previously described [31].

### 2.3. Pharmacokinetic modeling

Whole blood and intracellular pharmacokinetics of TAC were investigated using a non-parametric modeling approach (Pmetrics, version v. 1.5.2) [32]. The model was derived from the structural model previously developed by Robertsen *et al*. to describe everolimus concentrations in whole blood and PBMC [33]. Influence of covariates on individual pharmacokinetic parameters were investigated by multiple linear regression and non-parametric Kruskal-Wallis or Mann-Whitney comparison after graphical inspection. Thus, age, sex, body weight, albumin, hematocrit and count of PBMC in whole blood, were investigated as biological covariates. Additionally, SNPs in genes of *ABCB1*, *CYP3A4* and *CYP3A5* of donor and recipient were evaluated as genetic covariates of pharmacokinetic parameters. After graphical inspection, if significant association was found (p<0.01), covariates were individually introduced in the model. Decision to keep a covariate in the final model was based on the comparison of the Akaike information criterion (AIC), improvement of the bias and precision of the model. The model performance was assessed by diagnostic plots. An internal validation was performed using the visual predictive check (VPC) based on 1000 Monte-Carlo simulations.

### 2.4. Genotyping analysis

Genomic DNA was extracted from whole blood using a Janus automated workstation varispan (PerkinElmer, Courtaboeuf, France). Donor DNA was obtained from a DNA bank from the French Blood Agency. Genotyping were performed using Taqman® allelic discrimination assays (Thermofisher Waltham, MA, USA) on ABI 7900HT instrument (Applied Biosystems, Foster City, CA, USA).

Recipients and donors genotypes were determined for *CYP3A4* rs35599367 C>T (CYP3A4*22allele), *CYP3A5* rs776746 A>G (CYP3A5*3 allele). In addition, recipients and donor DNA were analyzed for the four SNPs of *ABCB1*: c.3435C>T (exon 26, rs1045642), ABCB1 c.1236 C>T (exon 12, rs1128503), ABCB1 c.2677 G>T/A (exon 21, rs2032582), and c.1199 G>A (exon 11, rs2229109).

### 2.5. Assessment of tacrolimus exposure in whole blood and PBMC and calcineurin activity assay

Whole-blood and intra-PBMC TAC concentrations were measured with a fully validated liquid chromatography tandem mass spectrometry procedure adapted from previously published methods [34,6]. Exposure to TAC was assessed in whole blood and PBMC by the areas under the curve of concentrations (AUC_0-12h_). AUC_0-12h_ were derived from the population pharmacokinetic model developed. Additionally, trough concentrations (C_0_), TAC peak concentrations (C_max_) and the times to peak concentration (T_max_) in whole blood and PBMC were extracted from the data set.

The activity of CaN in PBMC was measured by HPLC-Ultraviolet according to Blanchet *et al*. [35]. For the pharmacokinetic-pharmacodynamic analysis, CaN activity was expressed by the area under the activity (AUA_0-12h_) calculated by the trapezoidal method. Maximal inhibition of CaN on the inter-dose was calculated relatively to the basal CaN activity of the patient measured in PBMC on a blood sample collected just before the first TAC intake.

### 2.6. Probability of target attainment

The population pharmacokinetic model was applied to further investigate the pharmacokinetic-pharmacodynamic relationship of TAC by simulations. The probability of attainment of a target intracellular concentration of TAC inhibiting CaN activity (IC_target_), was explored for several ranges of TAC trough concentrations in whole blood. Ranges were selected according to TDM recommendations in liver transplanted patients. Multimodal Monte Carlo simulations were performed to generate 1,000 concentration time profiles per subject of the dataset. For each profile, trough whole blood concentrations (C0_WB_) were extracted and categorized in one of the C0_WB_ concentration range groups: 0–4 ng/mL (very low exposure), 4–6 ng/mL (low exposure), 6–10 ng/mL (recommended exposure). For each C0_WB_, the corresponding intra-PBMC C_max_ was estimated with the model. Then, the probability of achieving an intra-PBMC C_max_ above the IC_target_ was assessed for each C0_WB_ range.

### 2.7. Statistical analysis

Statistical analysis was performed using R software (version 3.2.5). Results are reported as means +/- standard error of the mean (SEM) or median and range. Shapiro-test was used to check variable distribution normality. Correlations between whole blood and intracellular pharmacokinetic parameters were performed using Pearson or Spearman test as appropriate. Coefficient of determination (r^2^) were reported and calculated from the corresponding coefficient of correlation when relationships were assessed by linear regression. The « SNPassoc package » was used to assess the Hardy-Weinberg equilibrium and “haplo.stat” to infer the most probable haplotype of *ABCB1 (3435/1236/2677) (*Package available on https://cran.r-project.org). Associations between TAC exposure parameters and genotypes were investigated using the non-parametric Mann-Whitney test or the Kruskal-Wallis test as appropriate. When a post-hoc test was required, the Bonferroni correction was applied.

Relationships between TAC concentrations in blood or PBMC and CaN activity were explored using linear regression. When needed, data were log-transformed and Shapiro-Wilk-test was applied to check normality of the residual of the linear model. Additionally, influence of TAC concentrations on CaN activity was analyzed with an inhibition model computed in Graphpad Prim (Version 8). A p-value< 0.05 was considered significant.

## 3. Results

### 3.1. Patients’ characteristics

A total of 32 liver transplant recipients were included in the study. Patients’ demographic and clinical characteristics are summarized in Table 1.

**Table 1:** Patients characteristics (n = 32).

**Demographic characteristics**	**Median [range] or n (%)**
Sex (M)	30 (94)
Age (years)	62 [51–70]
Body weight (kg)	97 [50–121]
Delay since transplantation (days)	9 [7–11]
**Biological characteristics (the day of the study)**	
Albumin (g/L)	23.8 [23.0–39.6]
Hematocrite (%)	30.5 [22.8–39.1]
PBMC count in whole blood (G/L)	2.3 [1.3–3.6]

M: masculine, PBMC: peripheral blood mononuclear cells

Delay since transplantation means time between transplantation and the day of the study.

### 3.2. Model development and validation

The model developed was a two compartments model, with two absorption phases described by a double gamma distribution in the first compartment. Other absorption models including lag time and two mean absorption times in the structural model were also assessed but lead to worse concentration predictions. Several polynomial error models were tested. The standard deviation (SD) of the TAC concentrations (C) representing the assay error was calculated by the formula SD = 1+0.1 x C for whole blood concentrations corresponding to an additive (in μg/L) and proportional (%) part of the assay error, respectively, and SD = 5+0.12 x C for PBMC concentrations. In addition, both concentrations were weighted by an additional noise term λ = 1, according to the formula: 1/(SD+λ)^2^. The population pharmacokinetic parameters are presented in S1 Table.

**Fig 1:**
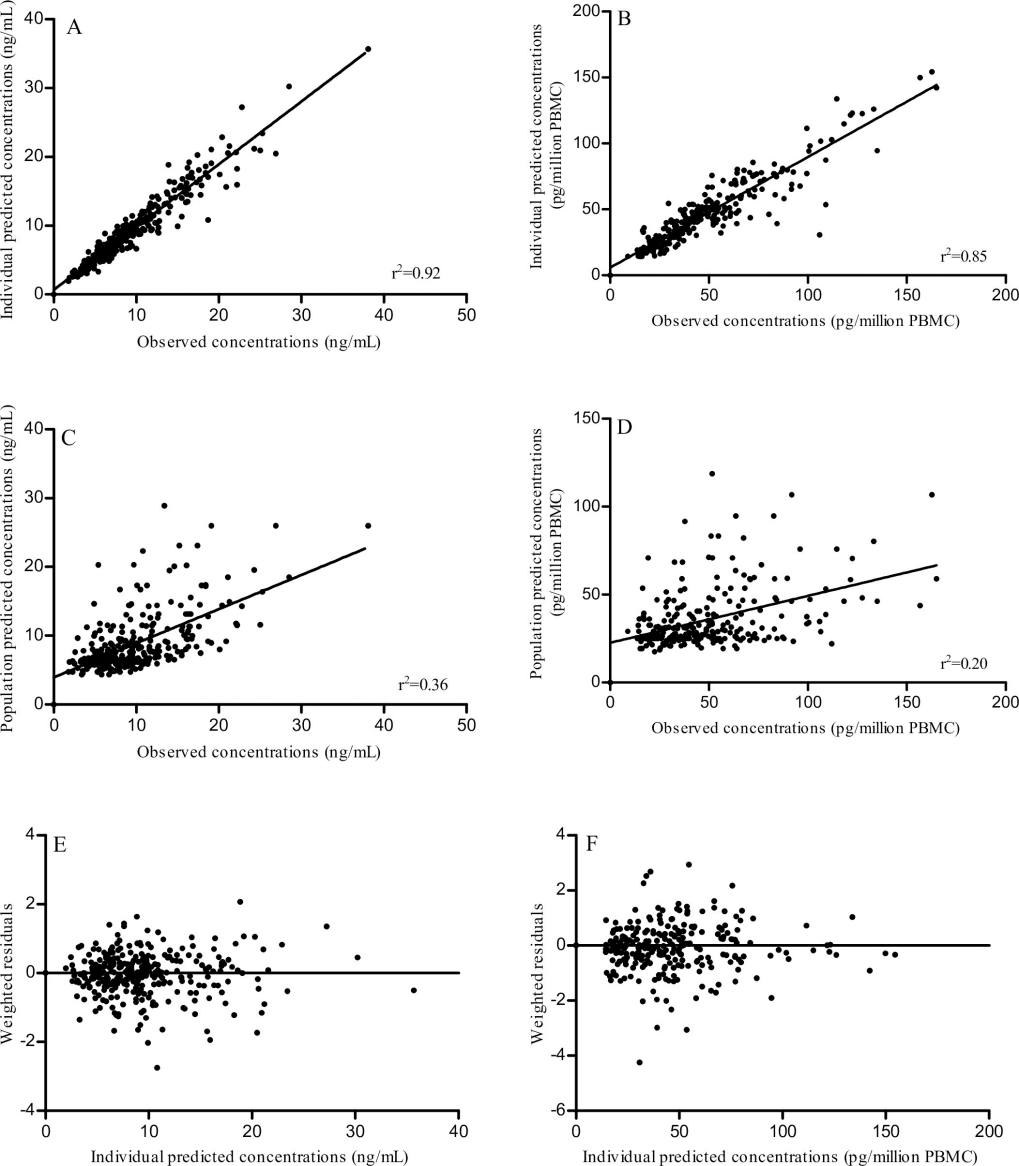
Model performances-diagnostic plots. Individual predicted versus observed concentrations of tacrolimus in whole blood (A) and in PBMC (B), population predicted versus observed concentrations of tacrolimus in whole blood (C) and in PBMC (D), weighted residuals versus individual predicted concentrations of tacrolimus in whole blood (E) and in PBMC (F).

Influences of covariates on model parameters were assessed. Significant associations were found between the count of PBMC in whole blood and the inter-compartmental rate constant k21 (p = 0.00354) and, between genotype 1199GA for rs2229109 (*ABCB1* exon 11) and C01 (model estimated whole blood trough concentration) (p = 0.00082) (S2 Table). Introduction of these covariates in the model did not improve the AIC neither the model precision. Plots of the model performances, for TAC in whole blood and in PBMC, are presented in Fig 1 as individual predicted versus observed plots, population predicted versus observed plots, weighted residuals versus predicted concentrations plots. The best and the worst fit of individual pharmacokinetic profiles with all-time points are reported in S1A and S1B Fig. Goodness of fit plots did not show any major bias in whole blood whereas a slight under-estimation was observed in PBMC. The weighted residuals were homogeneously distributed over the concentration ranges. For whole blood and PBMC, the VPC demonstrated that the majority of the normalized observed data fell within the 90% prediction intervals of the simulations and that the median tracks the middle of the observed data (S1C and S1D Fig).

### 3.3. Tacrolimus whole blood and intracellular pharmacokinetics

Time course profiles of TAC concentrations in whole blood and PBMC over 12h are presented in S2 Fig. Pharmacokinetic parameters of TAC in whole blood and PBMC are reported in Table 2.

**Table 2:** Tacrolimus pharmacokinetics parameters in whole blood and PBMC (n = 32).

	Median [range]	Mean (SD)
Dose (mg/12 h)	1.5 [0.5–4]	1.8 (1.0)
Dose (mg/kg/12h)	0.017 [0.005–0.048]	0.021 (0.012)
**Whole blood pharmacokinetics (WB)**		
C_max_ (ng/mL)	17.7 [3.5–36.3]	16.4 (6.9)
C_max_/dose (ng/mL/mg)	9.5 [3.0–21.4]	10.8 (5.2)
T_max_ (h)	1.6 [0.2–6]	1.9 (1.4)
C_0_ (ng/mL)^a^	6.2 [2.5–10.0]	6.4 (2.2)
C_0_/dose (ng/mL/mg)	3.9[1.1–17.7]	5.1 (4.2)
AUC_0–12h_ (ng∙h/mL)	102.3 [35.0–215.5]	108.9 (38.9)
Cl/F (L.h^-1^)	16.2 [5.0–39.2]	17.8 (9.0)
I**ntracellular pharmacokinetics (PBMC)**		
C_max_ (pg/million PBMC)	71.3 [25.7–156.0]	78.1 (37.1)
C_max_/dose (pg/million PBMC/mg)	44.2[17.1–258.7]	56.1 (46.1)
T_max_ (h)	1.6 [0.3–6]	1.9 (1.2)
C_0_ (pg/million PBMC)	28.4 [9.6–80.4]	37.2 (17.7)
C_0_/dose (pg/million PBMC/mg)	20.0 [3.2–67.2]	24.8 (16.8)
AUC_0–12h_ (pg∙h/million PBMC)	491.6 [223.0–1127.2]	
**Whole blood—intracellular relationships**		
Intracellular diffusion ratio (AUC_0–12h_ PBMC / AUC_0–12h_ WB)	23.6 [14.8–39.7]	24.9 (6.9)
	**r**^**2**^ **(p)**	
Correlation AUC_0–12h_ PBMC & AUC_0–12h_ WB	0.51 (<0.001)	
Correlation C_0_PBMC & C_0_WB	0.39 (<0.001)	
Correlation C_max_PBMC & C_max_WB	0.53 (<0.001)	
Correlation C_max_PBMC & C_0_WB	0.17 (0.02)	
Correlation AUC_0–12h_ PBMC & C_0_WB	0.53 (<0.001)	

C_max_: maximum concentration; T_max_: time when C_max_ is achieved; C_0_: predose concentration; AUC_0-12h_: area under the concentration–time curve from 0h to 12h; Cl/F: apparent clearance; PBMC: peripheral blood mononuclear cells; WB: whole blood; SD: standard deviation; r^2^: coefficient of determination; p: p-value

a: the day of the study, 81.2% of patients had tacrolimus whole blood concentration between 4–10 ng/mL.

Relationships between TAC AUC_0-12h_ and C_0_ or C_max_ were explored within each compartment. Significant but poor correlations were found between C_0_ and AUC in whole blood (r^2^ = 0.42, p<0.001) and in PBMC (r^2^ = 0.61, p<0.001). A weak correlation was found between C_max_ and AUC in whole blood (r^2^ = 0.52, p<0.001) and in PBMC as well (r^2^ = 0.55, p<0.001).

**Fig 2:**
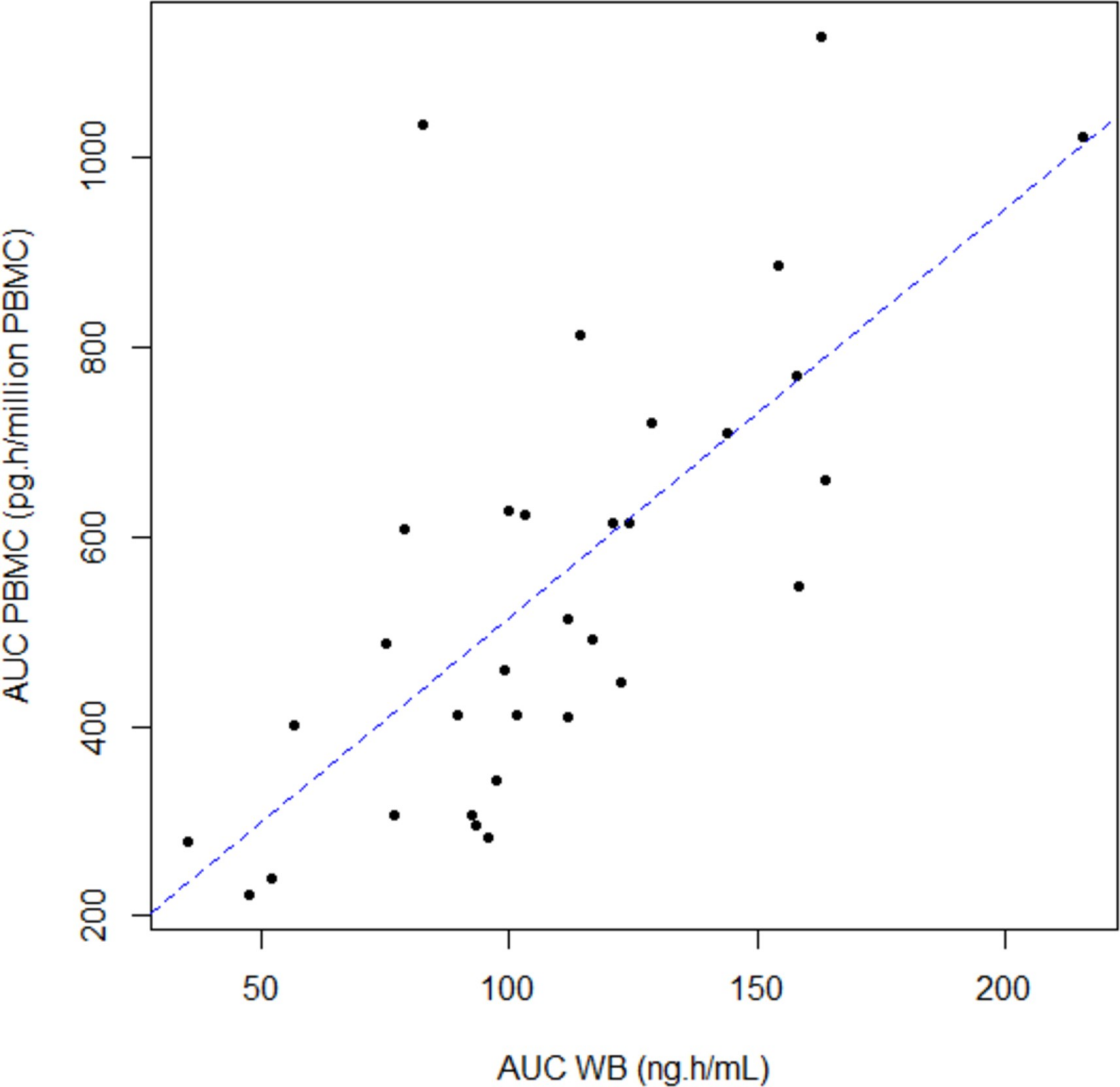
Relationship between area under the concentration–time curve from 0 to 12 h (AUC) of tacrolimus in whole blood (WB) and in peripheral mononuclear blood cells (PBMC). The dotted line is the linear regression curve. (n = 32) (r^2^ = 0.51, p<0.001).

Median intracellular distribution ratio of TAC (AUC_PBMC_/AUC_WB_) was 23.6. TAC exposure in PBMC was correlated to TAC exposure in whole blood. However, the strength of the linear association was weak, whatever the exposure parameters studied (r^2^< 0.53) (Table 2). Fig 2 displays the correlation between AUC_0-12h_ in whole blood and in AUC_0-12h_ in PBMC compartments.

No significant association was found between whole blood or PBMC pharmacokinetics parameters (AUC_0-12h_ and C_0_ or C_max_, AUC_PBMC_/AUC_WB_), and patients’ demographic characteristics (age, sex weight and time since transplantation). In addition, intra-PBMC exposure and intracellular distribution ratio were not correlated to serum albumin nor PBMC count in blood (p>0.05). Hematocrit appeared to have a slight influence on intra-PBMC exposure (AUC_0-12h_) and the distribution ratio (r = -0.31, p = 0.047 and r = -0.34, p = 0.036 respectively). Nevertheless, hematocrit value was not influenced by the time post transplantation (p = 0.13, Anova- test).

### 3.4. TAC pharmacokinetic-pharmacogenetic relationship

**Fig 3:**
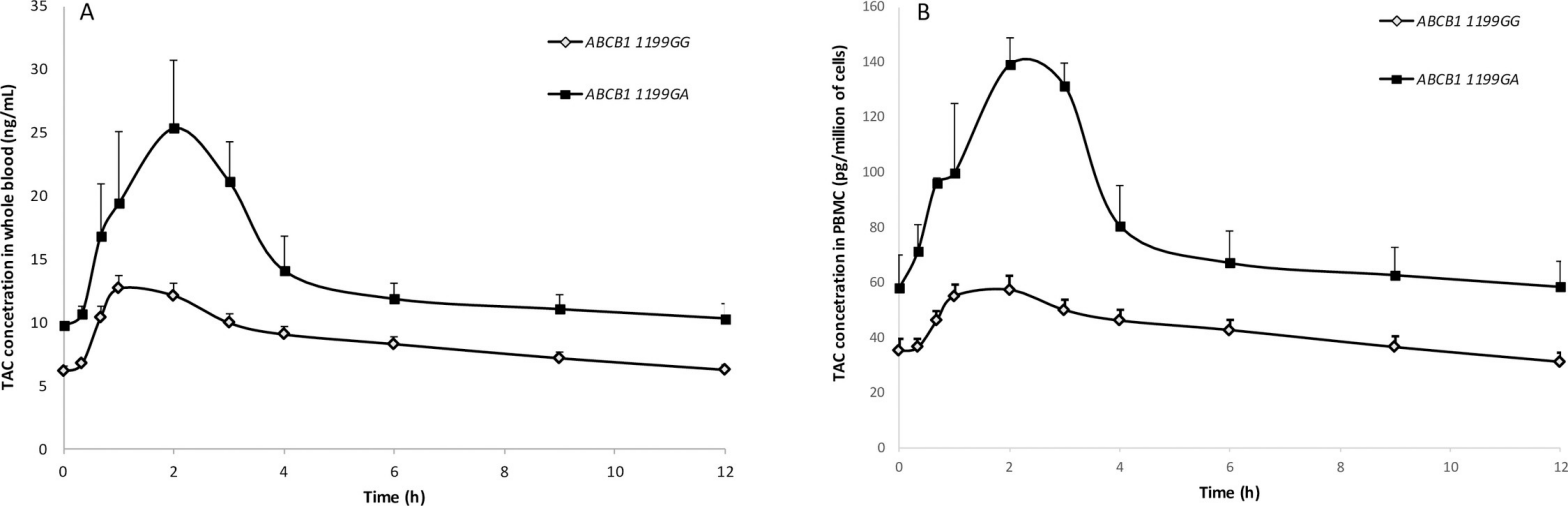
Influence of recipient *ABCB1* 1199G>A on whole blood and on intracellular (PBMC) areas under the tacrolimus (TAC) concentrations–time curve from 0 to 12 h (AUC). Each symbol represents mean ± standard deviation of the mean. (n = 29 *ABCB1* 1199GG, n = 3 *ABCB1* 1199GA).

Although integration of genetic covariates did not improve the pharmacokinetic model, the influences of SNPs on TAC exposure parameters in whole blood and PBMC were investigated. Genotypes frequencies for *ABCB1*, *CYP3A4* and *CYP3A5* (donor and recipient) genes are shown in Table 3. Hardy–Weinberg equilibrium was verified for each genotype except recipient *ABCB1* 3435C>T (exon 26, rs1045642) and donor *CYP3A5* rs776746 A>G. This deviation was attributed to the small size of our dataset since genotyping analysis of this SNP was double-checked by another external laboratory. Effect of each SNP on TAC pharmacokinetics in whole blood and PBMC are presented in Table 3. No significant association was found between *ABCB1* 3435C>T SNP and TAC pharmacokinetics in whole blood or PBMC. Recipients heterozygous CT for *ABCB1* 1236C>T polymorphism seemed to have lower AUC_0-12h_ and C_max_ in whole blood than wild type CC (p = 0.045 and p = 0.035 respectively), however these associations were not significant after Bonferroni correction (p = 0.092 and p = 0,107 respectively). Similarly, a lower whole blood C_max_ was observed in recipients heterozygous CT for *ABCB1* 2677G>T SNP (p = 0.046) but the difference compared to GG genotype was not significant after post-hoc analysis. Intracellular distribution ratio was lower in recipients homozygous for *ABCB1* 2677TT compared to 2677GT (p = 0.026). The ratio was also influenced by *ABCB1* haplotype (3435/1236/2677) since homozygous TTT/TTT had lower ratio than subject carrying only one TTT allele (p = 0.001). The SNP *ABCB1*1199G>A in recipient influenced both whole blood and intra-PBMC exposure. Median AUC_0-12h_, C_0_ and C_max_ were significantly higher among subjects carrying the “A” allele (p = 0.009, p = 0.009, p = 0.03 in whole blood, and p = 0.006, p = 0.04, p = 0.0008 in PBMC, respectively). This is illustrated in Fig 3 by whole blood and PBMC concentration versus time profiles according to recipient 1199G/A genotype. No influence of donor (graft) *ABCB1* genotype was found on TAC whole blood or PBMC exposures. A non-significant trend to lower C_0_ was observed in whole blood and PBMC for subject with a graft expresser of CYP3A5 (carrier of at least one 1 allele *1). Neither donor nor recipient CYP3A4*22 polymorphism had any impact on TAC pharmacokinetics in whole blood or PBMC.

**Table 3:** Influence of single nucleotide polymorphisms on TAC pharmacokinetics in whole blood and in PBMC.

Genotype	Allelic status	n	(%)	Tac AUC_0-12h WB_	Tac AUC_0-12h PBMC_	AUC_0-12h PBMC_/	Tac C_0 WB_	Tac C_0 PBMC_	Tac C_max WB_	Tac C_max PBMC_
				(ng.h/mL)	(pg.h/million of PBMC)	AUC_0-12h WB_	(ng/mL)	(pg/million of cells)	(ng/mL)	(pg/million PBMC)
Recipient ABCB1 3435C>T (rs1045642)	**CC**	2	(6)	132.2	769.1	27.4	6.7	49.9	19.4	101.2
				[101.4–162.9]	[411.1–1127.2]	[20.2–34.6]	[3.7–9.8]	[19.4–80.4]	[15.9–22.9]	[60.1–142.3]
	**CT**	21	(66)	99.7	548.8	24.3	6.2	30	15.3	71.3
				[35.0–215.5]	[223.0–1022.0]	[14.8–39.7]	[2.5–10.0]	[9.6–76.9]	[3.5–36.3]	[25.7–158.0]
	**TT**	9	(28)	103.1	486.6	19.2	6.2	23.2	18.3	74.9
				[75.1–163.8]	[295.7–1034.2]	[15.9–32.4]	[3.7–9.3]	[19.9–43.6]	[7.0–24.9]	[34.2–152.5]
Recipient ABCB1 1236 C>T (rs1128503)	**CC**	10	(31)	128.9	642	23.6	8.1	31.4	19.3	77.9
				[47.3–215.5]	[223.0–1127.2]	[14.8–35.6]	[3.7–9.8]	[9.6–80.4]	[5.9–36.3]	[25.7–156.8]
	**CT**	15	(47)	89.7	491.6	26.7	6.5	29.2	11	62.8
				[35.0–154.2]	[238.9–1034.2]	[17.6–39.7]	[2.5–10.0]	[17.7–60.9]	[3.5–22.4]	[28.9–158.0]
	**TT**	7	(22)	111.8	447.4	18.3	5.4	23.9	18.1	71.3
				[92.4–158.1]	[295.7–769.5]	[15.9–24.8]	[3.7–10.0]	[19.9–58.2]	[14.9–24.9]	[34.2–101.7]
Recipient ABCB1 2677 G>T/A (rs2032582)	**GG**	11	(34)	114	623.9	23.6	7.8	23.2	18.3	77
				[47.3–215.5]	[223.0–1127.2]	[14.8–35.6]	[3.7–9.8]	[9.6–80.4]	[5.9–36.3]	[25.7–156.8]
	**GT**	15	(47)	89.7	514.2	26.7	6.5	32.1	11.2	69.4
				[35.0–158.1]	[238.9–1034.2]	[20.0–39.7]	[2.5–10.0]	[17.7–60.9]	[3.5–22.4]	[28.9–158.0]
	**TT**	6	(19)	105.4	428.2	18.3	5.3	23.1	19.6	70
				[92.4–123.9]	[295.7–613.6]	[15.9–24.8] *	[3.7–6.2]	[19.9–43.9]	[17.5–24.9]	[34.2–101.7]
Recipient ABCB1 Haplotype 3435/1236/2177	**Het TTT**	17	(53)	99.1	514.2	25.1	6.2	32.1	14.9	69.4
				[35.0–158.1]	[238.9–1034.2]	[20.0–39.7]	[2.5–10.0]	[17.7–60.9]	[3.5–22.4]	[29.0–158.0]
	**HomTTT**	4	(13)	102.4	357.4	17.4	4.5	21.6	20.4	64.3
				[92.4–122.6]	[295.7–447.4]	[15.9–18.3] *	[3.7–6.2]	[19.9–23.9]	[17.5–24.9]	[34.2–82]
	**Other**	11	(34)	114	623.9	23.6	7.8	23.2	18.3	77.0
				[47.3–215.5]	[223.0–1127.2]	[14.8–35.6]	[3.7–9.8]	[9.6–80.4]	[5.9–36.3]	[25.7–156.8]
Recipient ABCB1 1199 G>A (rs2229109)	**GG**	29	(91)	99.7	486.6	23.1	6	23.9	17.5	69.4
				[35.0–163.8]	[223.0–1034.2]	[14.8–39.7]	[2.5–10.0]	[9.6–76.9]	[3.5–24.9]	[25.7–152.5]
	**GA**	3	(9)	162.9	1022	28	9.8	54.6	22.9	156.8
				[128.5–215.5]*	[720.4–1127.2]*	[23.7–34.6]	[9.6–10.0]*	[39.3–80.4]*	[19.9–36.3]*	[142.3–158.0]*
Recipient CYP3A4 (C>T) (*22, rs35599367)	**CC**	30	(94)	102.3	531.5	23.6	6.3	29.6	17.7	70.3
				[35.0–215.5]	[223.0–1127.2]	[14.8–39.7]	[2.5–10.0]	[9.6–80.4]	[3.5–36.3]	[25.7–158.0]
	**CT**	2	(6)	84.2	405.7	26.9	4.2	22.7	20.1	76.6
				[56.7–111.8]	[402.2–409.1]	[18.3–35.5]	[3.7–4.7]	[22.0–23.3]	[15.3–24.9]	[71.3–82.0]
Recipient CYP3A5 6986 G>A (*3, rs776746)	**AA**	32	(100)	102.3	502.9	23.6	6.2	28.4	17.7	71.3
				[35.0–215.5]	[223.0–1127.2]	[14.8–39.7]	[2.5–10.0]	[9.6–80.4]	[3.5–36.3]	[25.7–158.0]
Donor ABCB1 3435C>T (rs1045642)	**CC**	8	(25)	97.7	357.9	20.1	5.9	22.3	17.9	72.1
				[52.0–163.8]	[238.9–720.4]	[14.8–31.5]	[2.9–10.0]	[9.6–39.3]	[8.6–24.9]	[33.4–158.0]
	**CT**	12	(38)	96.1	453.7	24.3	5.1	28.5	15.6	71.3
				[35.0–158.1]	[277.6–1034.2]	[15.9–39.7]	[2.5–10.0]	[17.7–76.9]	[3.5–23.2]	[28.9–129.4]
	**TT**	12	(38)	120.3	581.2	24.2	7.2	34.8	19.9	74.2
				[47.3–215.5]	[223.0 1127.1]	[17.4–34.6]	[3.7–9.8]	[18.1–80.4]	[5.9–36.3]	[25.7–156.8]
Donor ABCB1 1236 C>T (rs1128503)	**CC**	11	(34)	111.7	514.2	23.0	5.8	23.9	18.3	74.9
				[35.0–163.8]	[238.9–813.0]	[14.8–39.7]	[2.5–10.0]	[9.6–76.9]	[3.5–24.9]	[28.9 158.0]
	**CT**	13	(41)	97.5	447.4	21.6	6.5	23.3	15.9	71.3
				[56.7–158.2]	[295.7–1034.2]	[15.9–35.5]	[3.7–10.0]	[18.1–58.2]	[7.0–23.3]	[40.5–152.5]
	**TT**	8	(25)	120.3	610.7	24.2	6.1	37.0	19.4	83.5
				[47.3–215.5]	[223.0–1127.2]	[21.1–38.6]	[3.7–9.8]	[19.2–80.4]	[5.9–36.3]	[25.7–156.8]
Donor ABCB1 2677 G>T/A (rs2032582)	**GG**	10	(31)	105.6	564.4	24.2	6.4	28.0	17.9	72.1
				[35.0–163.8]	[238.9–813.0]	[14.8–39.7]	[2.5–10.0]	[9.6–76.9]	[3.5–22.4]	[28.9–158.0]
	**GT/A**	11	(34)	97.48	411.1	20.1	4.9	22.2	17.6	74.9
				[56.7–143.8]	[295.7–1034.2]	[15.9–35.5]	[3.7–9.2]	[18.1–51.5]	[8.9–24.9]	[40.5–152.5]
	**TT/A**	11	(34)	123.9	607.8	24.3	7.0	43.6	18.1	65.4
				[47.3–215.5]	[223.0–1127.2]	[17.4–38.6]	[3.7–10.0]	[19.2–80.4]	[5.9–36.3]	[25.7–156.8]
Donor ABCB1 Haplotype 3435/1236/2177	**HetTTT**	14	(44)	98.3	473.3	23.2	5.8	28.5	16.8	68.3
				[56.7–158.2]	[295.7–1034.2]	[15.9–38.6]	[3.7–10.0]	[18.1–58.2]	[7.0–23.3]	[40.5–152.5]
	**HomTTT**	6	(19)	139.1	750.2	24.2	7.7	29.6	21.0	114.0
				[47.3–215.5]	[223.0–1127.2]	[21.1–34.6]	[3.7–9.8]	[19.2–80.4]	[5.9–36.3]	[25.7–156.8]
	**other**	12	(38)	105.7	461.7	23.0	6.1	23.2	17.9	72.1
				[35.0–163.8]	[238.7–813.0]	[14.8–39.7]	[2.5–10.0]	[9.6–76.9]	[3.5–24.9]	[28.9–158.0]
Donor ABCB1 1199 G>A (rs2229109)	**GG**	31	(97)	103.1	514.2	23.4	6.2	29.2	17.7	71.3
				[47.3–215.5]	[223.0–1127.2]	[14.8–38.6]	[3.0–10.0]	[9.6–80.4]	[5.9–36.3]	[25.7–158.0]
	**GA**	1	(3)	35.0 [n/a]	277.6 [n/a]	39.7 [n/a]	2.5 [n/a]	17.7 [n/a]	3.5 [n/a]	28.9 [n/a]
Donor CYP3A4 (C>T) (*22, rs35599367)	**CC**	27	(84)	99.7	491.6	24.5	6.2	29.2	17.5	71.3
				[35.0–162.9]	[223.0–1127.2]	[14.8–39.7]	[2.5–10.0]	[9.6–80.4]	[3.5–24.9]	[25.7–158.0]
	**CT**	5	(16)	158.2	548.8	20.3	8.5	23.4	18.3	74.9
				[89.7–215.5]	[411.1–1022.0]	[17.4–23.7]	[3.7–10.0]	[19.4–54.6]	[15.9–36.3]	[60.1–156.8]
Donor CYP3A5 6986 G>A (*3, rs776746)	**Expressor GG and GA**	2-Jan	(3)/(6)	103.1	447.4	18.3	4.8	19.9	23.2	77
				[95.8–122.6]	[283.7–623.9]	[14.8–30.3]	[3.8–4.9]	[9.6–23.2]	[20.2–23.3]	[74.9–152.5]
	**Non expressor AA**	29	(91)	101.44	514.2	23.6	6.5	30	17.5	69.4
				[35.0–215.5]	[223.0–1127.2]	[15.9–39.7]	[2.5–10.0]	[17.7–80.4]	[3.4–36.2]	[25.72–158.0]

AUC_0-12h:_ area under the concentration–time curve from 0h to 12h; PBMC: peripheral blood mononuclear cells; WB: whole blood; C_0_: predose concentration; C_max_: maximum concentration; Het: heterozygote; Hom: homozygote. Data are expressed as median [range]

* p<0.05.

### 3.5. Tacrolimus pharmacokinetic-pharmacodynamic relationship

The time course profiles of CaN activity between two TAC intakes is shown in S2 Fig. Pharmacodynamic parameters related to CaN activity in PBMC are reported in Table 4. Median minimal CaN activity (CaN_min_) was achieved 2h post TAC intake (T_min_) which is slightly delayed after the T_max_ of TAC in whole blood and PBMC (1.6h). Coefficients of variation of pharmacodynamic parameters were higher than 30% which reflects a high inter-patient variability of CaN activity. No correlation was found between AUA_0-12h_ and TAC AUC_0-12h_ in whole blood or PBMC. Inhibition of CaN compared to its basal value (i.e. before TAC treatment) was never complete and the median CaN_Imax_ was only -37%.

**Table 4:** Pharmacodynamic parameters (n = 32).

	Median [range]	CV (%)
AUA_0-12h_ (pmol.h/min/million PBMC)	4864 [2903–8598]	36
CaN basal (pmol/min/million PBMC)	371.6 [151.6–1550.1]	66
CaN_min (0-12h)_ (pmol/min/million PBMC)	317.3 [96–547.8]	39
CaN_moy_ (pmol/min/million PBMC)	412.2 [229.9–791.96]	32
% Inhibition max	-37 [–74–50]	45
T_min_ (h)	2 [0.33–12]	n/a

AUA_0-12h_: area under the calcineurin-time curve from 0 to12h; CaN basal: calcineurin activity at baseline before the first administration of tacrolimus; CaNmin_(0-12h)_: calcineurin activity when inhibition is maximal between 0 and 12h post tacrolimus intake; CaN_moy_: mean calcineurin activity between 0 and 12h post tacrolimus intake; T_min_: Time corresponding to CaN_min (0-12h)_. PBMC: peripheral blood mononuclear cells CaN activity assay was based on the detection of the product formed by CaN incubated with a peptide substrate. CaN activity was expressed as picomoles of dephosphorylated peptides per minute per 10^6^ cells

Using a linear model, an association was found between CaN_Imax_ in PBMC and C_max_ of TAC (log-transformed) in PBMC (r^2^ = 0.21, p = 0.019) or in whole blood (r^2^ = 0.27, p = 0.007). The trend of the concentration dependent inhibition of CaN activity by TAC was better described using an equation like CaN_Imax_ = I_min_+(I_min_-I_max_)/(1+(C_max_/IC_50_)) where CaN_Imax_ is the maximal inhibition of CaN activity observed on the 0-12h period for a concentration C_max_ of TAC, I_min_ is the highest inhibitory effect and I_max_ is the lowest inhibitory effect. IC_50_ is TAC C_max_ which gives a 50% inhibition of CaN compared to basal activity.

**Fig 4:**
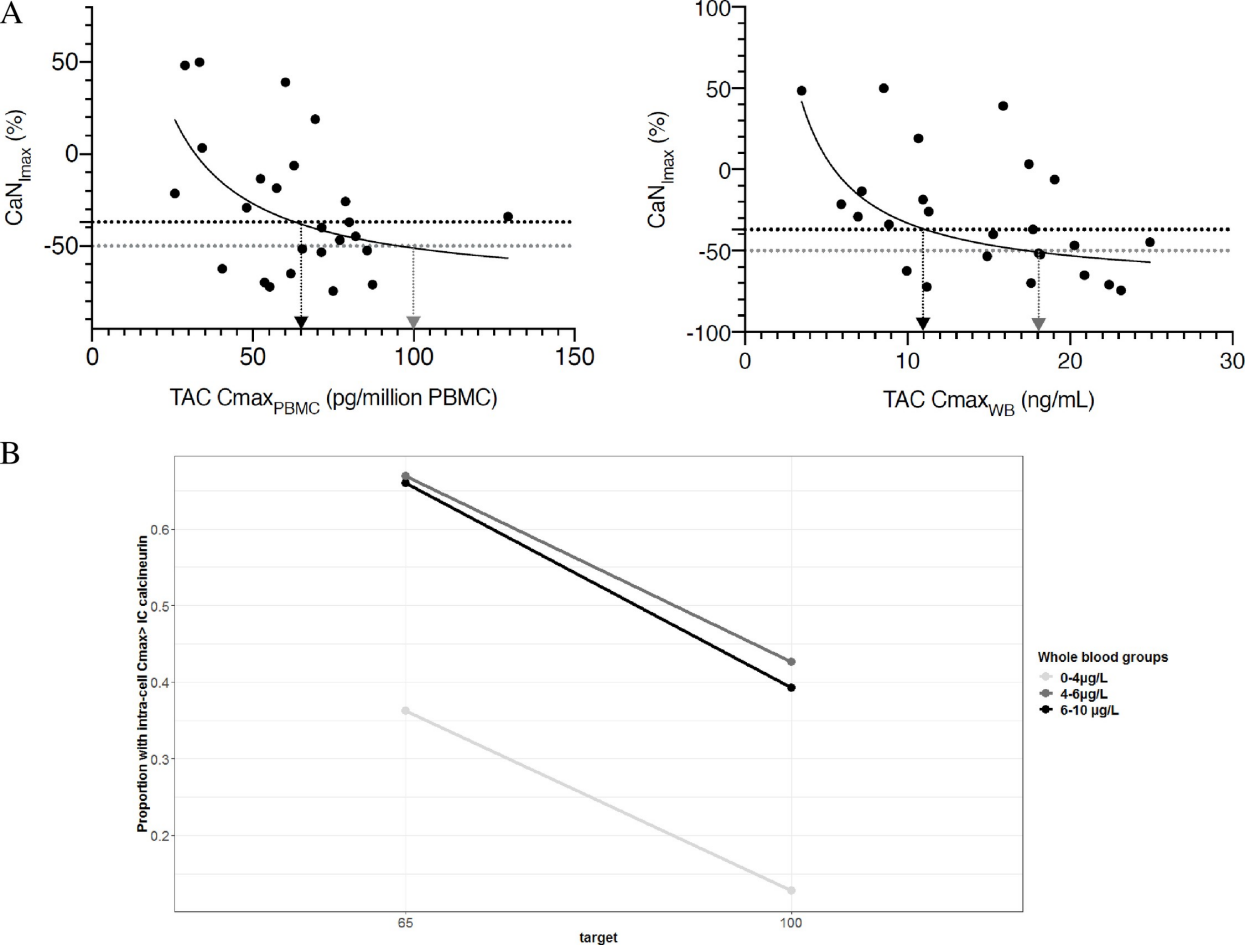
Tacrolimus (TAC) pharmacokinetic-pharmacodynamic relationship. (A): Relationship between calcineurin maximum inhibition (CaN_Imax_) and TAC maximum concentration (C_max_) in peripheral mononuclear cells (PBMC) or whole blood (WB). Black arrows show tacrolimus concentration inhibiting 37% (IC_37_) of calcineurin activity (65 pg/million of cells in PBMC and 11 ng/mL in whole blood) and greys arrows show tacrolimus concentration inhibiting 50% (IC_50_) of calcineurin activity (100 pg/million of cells in PBMC and 18 ng/mL in whole blood). (n = 32). Probability of intracellular target attainement (B). Targets are IC_37 and_ IC_50_ in PBMC. IC: Inhibitory concentration.

Graphically, the IC_50_ of TAC for CaN was 18 ng/mL in whole blood and 100 pg/million of cells in PBMC. In addition, the IC_37_ (TAC C_max_ which gives the median CaN_Imax_ of 37% in the study) was 11 ng/mL in whole blood and 65 pg/millions of cells in PBMC (Fig 4A).

Intra-PBMC inhibitory concentrations of CaN where used as IC_target_ of TAC C_max_ to explore the probability of target attainment of these intracellular concentrations for several ranges of trough whole blood concentrations. Less than 50% of patients were expected to achieve intra-PBMC IC_50_ whatever their C_0WB_ group (13%, 39% and 42% for the low, medium and high exposure groups respectively). The probability to achieve the intra-PBMC IC_37_ was 36% for the low exposure group, 66% for the medium exposure group and 67% for the group with highest C_0WB_ (Fig 4B).

## 4. Discussion

To the best of our knowledge, this is the first study aiming at exploring the complete pharmacogenetic-whole blood/intracellular pharmacokinetic-pharmacodynamic (PG-PK^2^-PD) relationship of TAC in liver transplant recipients. Most of the studies published to date aimed at exploring part of this relationship (i.e. PG-PK or PK-PD) but none had given a complete overview of this relationship in a quite large number of rich pharmacological profiles. These full profiles were used to model TAC concentrations in whole blood and, for the first time, in PBMC. The model quite well described the drug concentrations in both compartments. As observed on individual predicted versus observed plots, intra-PBMC concentrations were slightly under-estimated for a few patients. One can raise the hypothesis that it might be due to the normalization of TAC intra-PBMC concentration in quantity per number of cells instead of volumetric unit. Indeed, as suggested by Pensi *et al*., it would be more accurate to normalize intracellular concentration by the mean cell volume of each patient [7,36]. Unfortunately, measurement of cells volume was not available on the instrument use in our study and we could not check whether the underestimated concentrations were due to extreme values in PBMC volumes in these individuals.

Although it should be further validated in an independent cohort, the model appears to be a useful tool to assess TAC intracellular exposure. This is of particular interest since the experimental process to quantify TAC in cells is time-consuming and can be challenging for an application in routine practice.

One of the most point open for criticism of this study is our choice to assess TAC exposure in the early postoperative period which is thought to be unstable. In particular, hematocrit is assumed to change in the early phase post transplantation which could alter equilibrium between whole blood and intracellular TAC concentrations. However we consider that the study was performed in a relative steady state. Indeed, concentrations were measured beyond 7 days post-transplantation and TAC dose was unchanged since at least 3 days before blood collection. In addition, we showed that hematocrit was not significantly different within to the range of times post transplantation in our cohort. The poor correlation between hematocrit and intracellular exposure explains that the influence of this parameter was not sufficient to retain it as a significant covariate in the model developed. Moreover, we chose to focus on this early period post transplantation because acute rejection in liver recipients is more frequent within the first 10 postoperative days. It appears then much more relevant to study biomarkers related to TAC pharmacokinetics variability during this critical period.

In the present work, we provided a pharmacogenetics analysis to explore the influence of SNPs in both donors and recipients, on TAC pharmacokinetics in the early period post-transplantation. We focused on the most relevant polymorphisms which have been associated with TAC pharmacokinetics in the literature [13].

Interestingly, we found an impact of recipient *ABCB1* polymorphisms 1199G>A on whole blood but also on intracellular exposure of the drug. Despite the actual relevance of this result could suffer from the small sample size and should be confirmed further, it is consistent with previous reports. Indeed, it is assumed that the variant 1199A lead to a lower activity of ABCB1 protein. In a functional in-vitro study in recombinant cell line, Dessilly *et al*. showed that TAC efflux was strongly lower in cells expressing the variant allele (1199A) compared to wild type cells [21]. Thus, 1199A genotype in recipient could lead to increase whole blood exposure by increasing its absorption through the intestinal barrier (due to decreased TAC efflux), and to increase TAC accumulation in PBMC due to a less active efflux from the cells. This mechanism was confirmed in a clinical study from the same group showing that TAC PBMC concentration at day 7 post kidney transplantation was 1.4 fold higher in recipient carrier of the 1199A allele [18]. Besides, recipient *ABCB1* 2677TT genotype and haplotype (3435/1236/2677) influenced TAC blood to PBMC diffusion ratio leading to lower ratio in patient homozygous TTT. Conflicting results have been reported regarding *ABCB1* 2677G>T polymorphism or corresponding haplotype and TAC dose requirement [12,13,37,38]. In liver transplant recipient *ABCB1* haplotype is not expected to have a strong impact on TAC whole blood pharmacokinetics but it could significantly influence leukocytes concentration since P-gp is expressed in the cell membrane. Contrary to observations of TAC accumulation in hepatocytes for T variant carrier [20], in PBMC, presence of T-allele seemed to lower intracellular exposure. The molecular mechanism remains to be elucidated. Nevertheless, a limit of our study is the size of our data set which was likely not large enough to evidence accurately all genetic associations for SNP with low allele frequency. Then these results should be further confirmed in larger cohorts. Besides, we did not assess the influence of corticosteroids dosages on tacrolimus concentrations in whole blood and PBMC. It is unfortunate since steroids are known to induce CYP3A and ABCB1 expression so it could have been valuable to look for the influence of this covariate as well.

Besides, whole-blood and intracellular concentrations seems to be correlated contrary to what has been initially reported in a previous study [30]. This relationship legitimates TAC TDM in whole blood since whole blood concentrations could roughly reflect concentration in the target compartment. A work from Han *et al*. in kidney transplantation, exploring the relationship between whole blood and intracellular TAC concentrations, ended to the same conclusion [10]. However, whole blood concentrations are only a partial reflection of intracellular concentrations as highlighted by the mild to moderate correlations found between different time points (r^2^ ranging from 0.17 to 0.53). This poor association might not be due to the physiologic instability in the early period post transplantation. Indeed, a work from Klaasen *et al*. refutes the hypothesis of the lack of correlation linked to *de novo* status of the liver, since the authors reported remaining poor correlations between whole blood and intra-PBMC tacrolimus concentrations from 1 week to 1 year post transplantation [39]. As confirmed by the pharmacogenetic association study, drug transporters such as P-gp may at least partially explain these variabilities. This might also explain why some patients exhibit adverse events (rejection or toxicity) while having whole blood concentrations within the therapeutic range. New strategies aiming at evidencing this sup-population of patients displaying inconsistencies between whole blood concentrations and clinical outcome are needed. While confirmation of its value is required, measuring TAC intracellular concentrations might help detecting this at-risk population. Moreover, consistently with previous reports in the literature, poor correlations were observed between AUC of tacrolimus and trough concentrations within the same matrix, which shows that trough concentration is an imperfect surrogate marker of AUC [2,40].

Considering pharmacodynamic parameters, we report a low and relatively flat calcineurin inhibition in the study population. In addition, a relatively high inter-patient variability of CaN activities was observed. These results are consistent with previous published work on that topic [27–29]. We tried to develop a pharmacokinetic-pharmacodynamic model to describe the relationship between TAC exposure and inhibition of its molecular target but all modeling approaches tested failed. It was attributed to the size of our data set and the flat profile of CaN activity over time within patients. Moreover, another determinant of the complex relationship between CaN activity and exposure to TAC could be SNPs in the promoter region of the catalytic subunit of CaN (e.g. PPP3CA, rs45441997), as highlighted by the work of Noceti *et al*. [41]. Unfortunately, genetic analysis of gene involved in the pharmacodynamic pathway of TAC could not be performed in our work.

A non-complete inhibition of CaN was observed since the median CaN inhibition was 37%. High TAC whole blood concentrations have been previously reported as CaN IC_50_ (i.e 26.4 ng/mL for Fukudo *et al*. and 20.9 ng/mL for Yano *et al*.). These concentrations are hardly reached with modern TAC minimizing strategy [27,42]. In our study, we found an IC_50_ of 18 ng/mL for whole blood TAC concentration, a value slightly below but close to previous findings. More interestingly, we also highlighted an intracellular concentration of 100 pg/million cells as *in vivo* intracellular CaN IC_50_. This value is slightly lower than the one previously determined *in vitro* by our group (160 pg/million cells) [31]. Again, this threshold is rarely reached with current drug regimen in liver transplantation. When simulating 1,000 concentrations profiles and determining their intracellular C_max_, we observed that a few patients reached the concentration threshold corresponding to the IC_50_. When categorizing simulated patients according to their whole blood trough concentrations, patients with a C_0WB_ lower than 4 ng/mL almost never reach the intracellular concentration threshold for IC_50_ while roughly the same proportion of patients (but less than 50%) with C_0WB_ between 4 and 6 ng/mL or 6 and 10 ng/mL (i.e. the actual target recommendation in liver transplantation) reach the target. Despite not consistently attaining the threshold even with the current recommended trough whole blood concentrations (6–10 ng/mL), TAC based treatments seems to present sufficient efficacy in term of rate of rejection and graft survival. This means that, in the era of a combined immunosuppressive therapy associating TAC, mycophenolic acid or m-TOR inhibitor and corticosteroids, a high level of CaN inhibition may not be necessary. In agreement with this statement, Daher Abdi *et al*. reported that immunosuppressive efficacy (in renal transplant recipients) was not associated with calcineurin inhibitor exposure while mycophonolic acid exposure significantly mattered [43]. This hypothesis is emphasized by the median maximal CaN inhibition found in our study (37%). Considering this median CaN inhibition as a threshold, only 36% of patients in the very low exposure group of simulated patients would reach the corresponding intracellular C_max_ (65 pg/million cells) while difference in the proportion of patients reaching it in the low-exposure or recommended exposures groups were again similar (66% versus 67%).

The main limitation of our study is the lack of clinical endpoint to link with the PG-PK^2^-PD analysis. In particular, it should have been relevant to confront our results to toxicity or rejection rate. However, ACR monitoring could not be included in the design of the study since in our center liver biopsy is not systematically performed in the patient’s follow-up to diagnose ACR. Moreover, data regarding associations between genetic biomarkers and graft or patient survival are lacking but will be obtained from the larger CYPTAC’H study on going in our center.

## 5. Conclusion

In conclusion, a population pharmacokinetic model was successfully developed and applied to the first global investigation of the pharmacogenetic-whole blood/intracellular pharmacokinetic-pharmacodynamic (PG-PK^2^-PD) relationship of TAC in liver transplant recipients. Recipient *ABCB1* polymorphisms 1199G>A could influence whole blood but also intracellular exposure of TAC but the clinical relevance of this genetic variant remains to be investigated. In addition, CaN activity appeared incompletely inhibited by TAC and only few patients were expected to reach intracellular IC_50_ concentrations at therapeutic whole blood concentration suggesting alternative pharmacodynamic effects of TAC than CaN inhibition. These results should be confirmed in a larger cohort. Further studies are required to clarify the relationship between intracellular TAC exposure and clinical outcomes in order to find whether TAC intracellular concentration could be useful to tailor the immunosuppressive therapy.

## Supporting information

S1 Fig Model performances and validation. Worst (A) and best (B) individual predicted profiles for tacrolimus (TAC) in whole blood (bottom curve) and in peripheral blood mononuclear cells (PBMC) (upper curve). Green line represents observed tacrolimus concentrations in PBMC, red line represents fitting of the model for PBMC concentrations. Blue line represents observed tacrolimus concentrations in whole blood, black line represents fitting of the model for whole blood concentrations. Visual predictive checks for whole blood (C) and PBMC (D) concentration of tacrolimus. Grey zones are confidence intervals at 95% of 5th, 50th and 95th percentiles of predictions obtained from 1000 Monte-Carlo simulations from the model. Curves are percentiles of the observed data. These curves must be included within the confidence interval above mentioned.
(PDF)

S2 Fig **Time course profiles of tacrolimus (TAC) concentrations in whole blood and PBMC (left axis) and calcineurin activity in PBMC (right axis).** Each symbol represents mean ± standard deviation of the mean. TAC_WB_: tacrolimus concentration in whole blood, TAC_PBMC_: tacrolimus concentration in PBMC, CaN: calcineurin, PBMC: peripheral blood mononuclear cells. (n = 32).
(PDF)

S1 Table Population pharmacokinetics parameters. (DOCX)

S2 Table Combined genotypes frequencies. (DOCX)

S3 Table Association analysis between covariates and model parameters. (DOCX)

S4 Table Hardy-Weinberg equilibrium analysis. (DOCX)

## Abbreviations

ABCB1: ATP-binding cassette subfamily B member 1
ACR: acute cellular rejection
AUA: area under the calcineurin -time curve from 0 to12h
AUC_0-12h_: area under the concentration–time curve from 0h to 12h
C0: trough or predose concentration
CaN: calcineurin
CaN min: median minimal CaN activity
CaN_Imax_: calcineurin maximum inhibition
C_max_: maximum concentration
CYP3A4/5: cytochrome P450 isoform 3A4/5
DNA: deoxyribonucleic acid
EDTA: ethylene diamine tetracetique
HPLC: high performance liquid chromatography
IC50: concentration inhibiting 50% of the maximal activity of calcineurin
IC37: concentration inhibiting 37% of the maximal activity of calcineurin
IC_target_: target intracellular concentration of TAC inhibiting CaN activity
IL2: interleukin-2
PBMC: peripheral blood mononuclear cells
PD: pharmacodynamic
PG: pharmacogenetics
P-pg: P-glycoprotein
PK: pharmacokinetic
SD: standard deviation
SEM: standard deviation of the mean
SNP: single nucleotide polymorphism
TAC: tacrolimus
TDM: therapeutic drug monitoring
T_max_: time when C_max_ is achieved
T_min_: Time corresponding to CaNmin (0-12h).
VPC: visual predictive check

## References

[1] Thomson AW, Bonham CA, Zeevi A. Mode of action of tacrolimus (FK506): molecular and cellular mechanisms. Ther Drug Monit. déc 1995;17(6):584–91. 10.1097/00007691-199512000-00007 8588225

[2] Brunet M, van Gelder T, Åsberg A, Haufroid V, Hesselink DA, Langman L, et al Therapeutic Drug Monitoring of Tacrolimus-Personalized Therapy: Second Consensus Report. Ther Drug Monit. juin 2019;41(3):261–307. 10.1097/FTD.0000000000000640 31045868

[3] Staatz CE, Tett SE. Clinical Pharmacokinetics and Pharmacodynamics of Tacrolimus in Solid Organ Transplantation: Clinical Pharmacokinetics. 2004;43(10):623–53. 10.2165/00003088-200443100-00001 15244495

[4] Capron A, Lerut J, Latinne D, Rahier J, Haufroid V, Wallemacq P. Correlation of tacrolimus levels in peripheral blood mononuclear cells with histological staging of rejection after liver transplantation: preliminary results of a prospective study: PBMCs tacrolimus levels and graft rejection. Transplant International. janv 2012;25(1):41–7. 10.1111/j.1432-2277.2011.01365.x 21981711

[5] Capron A, Musuamba F, Latinne D, Mourad M, Lerut J, Haufroid V, et al Validation of a liquid chromatography-mass spectrometric assay for tacrolimus in peripheral blood mononuclear cells. Therapeutic drug monitoring. 2009;31(2):178–186. 10.1097/FTD.0b013e3181905aaa 19057467

[6] Lemaitre F, Antignac M, Fernandez C. Monitoring of tacrolimus concentrations in peripheral blood mononuclear cells: Application to cardiac transplant recipients. Clinical Biochemistry. oct 2013;46(15):1538–41. 10.1016/j.clinbiochem.2013.02.011 23454394

[7] Pensi D, De Nicolò A, Pinon M, Calvo PL, Nonnato A, Brunati A, et al An UPLC–MS/MS method coupled with automated on-line SPE for quantification of tacrolimus in peripheral blood mononuclear cells. Journal of Pharmaceutical and Biomedical Analysis. mars 2015;107:512–7. 10.1016/j.jpba.2015.01.054 25698619

[8] Capron A, Haufroid V, Wallemacq P. Intra-cellular immunosuppressive drugs monitoring: A step forward towards better therapeutic efficacy after organ transplantation? Pharmacol Res. sept 2016;111:610–8. 10.1016/j.phrs.2016.07.027 27468645

[9] Lemaitre F, Antignac M, Verdier M-C, Bellissant E, Fernandez C. Opportunity to monitor immunosuppressive drugs in peripheral blood mononuclear cells: Where are we and where are we going? Pharmacological Research. août 2013;74:109–12. 10.1016/j.phrs.2013.06.003 23792083

[10] Han SS, Yang SH, Kim MC, Cho J-Y, Min S-I, Lee JP, et al Monitoring the Intracellular Tacrolimus Concentration in Kidney Transplant Recipients with Stable Graft Function. PLoS ONE. 2016;11(4):e0153491 10.1371/journal.pone.0153491 27082871 PMC4833335

[11] Bahmany S, de Wit LEA, Hesselink DA, van Gelder T, Shuker NM, Baan C, et al Highly sensitive and rapid determination of tacrolimus in peripheral blood mononuclear cells by liquid chromatography-tandem mass spectrometry. Biomed Chromatogr. janv 2019;33(1):e4416. 10.1002/bmc.4416 PMC6587946 30362145

[12] Goto M, Masuda S, Kiuchi T, Ogura Y, Oike F, Tanaka K, et al Relation between mRNA expression level of multidrug resistance 1/ABCB1 in blood cells and required level of tacrolimus in pediatric living-donor liver transplantation. J Pharmacol Exp Ther. mai 2008;325(2):610–6. 10.1124/jpet.107.135665 18252812

[13] Staatz CE, Goodman LK, Tett SE. Effect of CYP3A and ABCB1 single nucleotide polymorphisms on the pharmacokinetics and pharmacodynamics of calcineurin inhibitors: Part I. Clin Pharmacokinet. Mars 2010;49(3):141–75. 10.2165/11317350-000000000-00000 20170205

[14] Picard N, Bergan S, Marquet P, van Gelder T, Wallemacq P, Hesselink DA, et al Pharmacogenetic biomarkers predictive of the pharmacokinetics and pharmacodynamics of immunosuppressive drugs. Therapeutic drug monitoring. 2016;38:S57–S69. 10.1097/FTD.0000000000000255 26469711

[15] Hesselink DA, Bouamar R, Elens L, van Schaik RHN, van Gelder T. The Role of Pharmacogenetics in the Disposition of and Response to Tacrolimus in Solid Organ Transplantation. Clinical Pharmacokinetics. Févr 2014;53(2):123–39. 10.1007/s40262-013-0120-3 24249597

[16] Haufroid V, Mourad M, Van Kerckhove V, Wawrzyniak J, De Meyer M, Eddour DC, et al The effect of CYP3A5 and MDR1 (ABCB1) polymorphisms on cyclosporine and tacrolimus dose requirements and trough blood levels in stable renal transplant patients. Pharmacogenetics. mars 2004;14(3):147–54. 10.1097/00008571-200403000-00002 15167702

[17] Tron C, Lemaitre F, Verstuyft C, Petitcollin A, Verdier M-C, Bellissant E. Pharmacogenetics of Membrane Transporters of Tacrolimus in Solid Organ Transplantation. Clin Pharmacokinet. mai 2019;58(5):593–613. 10.1007/s40262-018-0717-7 30415459

[18] Capron A, Mourad M, De Meyer M, De Pauw L, Eddour DC, Latinne D, et al CYP3A5 and ABCB1 polymorphisms influence tacrolimus concentrations in peripheral blood mononuclear cells after renal transplantation. Pharmacogenomics. Mai 2010;11(5):703–14. 10.2217/pgs.10.43 20415563

[19] Vafadari R, Bouamar R, Hesselink DA, Kraaijeveld R, van Schaik RH, Weimar W, et al Genetic polymorphisms in ABCB1 influence the pharmacodynamics of tacrolimus. Therapeutic drug monitoring. 2013;35(4):459–465. 10.1097/FTD.0b013e31828c1581 23743668

[20] Elens L, Capron A, Kerckhove VV, Lerut J, Mourad M, Lison D, et al 1199G&gt;A and 2677G&gt;T/A polymorphisms of ABCB1 independently affect tacrolimus concentration in hepatic tissue after liver transplantation: Pharmacogenetics and Genomics. 10 2007;17(10):873–83. 10.1097/FPC.0b013e3282e9a533 17885626

[21] Dessilly G, Elens L, Panin N, Capron A, Decottignies A, Demoulin J-B, et al ABCB1 1199G&gt;A Genetic Polymorphism (Rs2229109) Influences the Intracellular Accumulation of Tacrolimus in HEK293 and K562 Recombinant Cell Lines. Zhang J-T, éditeur. PLoS ONE. 12 Mars 2014; 9(3):e91555 10.1371/journal.pone.0091555 24621983 PMC3951418

[22] Woillard J-B, Gatault P, Picard N, Arnion H, Anglicheau D, Marquet P. A donor and recipient candidate gene association study of allograft loss in renal transplant recipients receiving a tacrolimus-based regimen. Am J Transplant. Déc 2018;18(12):2905–13. 10.1111/ajt.14894 29689130

[23] Birdwell KA, Decker B, Barbarino JM, Peterson JF, Stein CM, Sadee W, et al Clinical Pharmacogenetics Implementation Consortium (CPIC) Guidelines for CYP3A5 Genotype and Tacrolimus Dosing. Clin Pharmacol Ther. Juill 2015;98(1):19–24. 10.1002/cpt.113 25801146 PMC4481158

[24] Brunet M, Shipkova M, van Gelder T, Wieland E, Sommerer C, Budde K, et al Barcelona consensus on biomarker-based immunosuppressive drugs management in solid organ transplantation. Therapeutic drug monitoring. 2016;38:S1–S20. 10.1097/FTD.0000000000000287 26977997

[25] Sanquer S, Amrein C, Grenet D, Guillemain R, Philippe B, Boussaud V, et al Expression of Calcineurin Activity after Lung Transplantation: A 2-Year Follow-Up. Gregson A, éditeur. PLoS ONE. 25 Mars 2013;8(3):e59634 10.1371/journal.pone.0059634 23536885 PMC3607585

[26] Fukudo M, Yano I, Katsura T, Ito N, Yamamoto S, Kamoto T, et al A transient increase of calcineurin phosphatase activity in living-donor kidney transplant recipients with acute rejection. Drug Metab Pharmacokinet. 2010;25(5):411–7. 10.2133/dmpk.dmpk-10-rg-026 20834189

[27] Yano I, Masuda S, Egawa H, Sugimoto M, Fukudo M, Yoshida Y, et al Significance of trough monitoring for tacrolimus blood concentration and calcineurin activity in adult patients undergoing primary living-donor liver transplantation. European Journal of Clinical Pharmacology. Mars 2012;68(3):259–66. 10.1007/s00228-011-1129-x 21969228

[28] Blanchet B, Duvoux C, Costentin CE, Barrault C, Ghaleh B, Salvat A, et al Pharmacokinetic-pharmacodynamic assessment of tacrolimus in liver-transplant recipients during the early post-transplantation period. Therapeutic drug monitoring. 2008;30(4):412–418. 10.1097/FTD.0b013e318178e31b 18641556

[29] Iwasaki M, Yano I, Fukatsu S, Hashi S, Yamamoto Y, Sugimoto M, et al Pharmacokinetics and Pharmacodynamics of Once-Daily Tacrolimus Compared With Twice-Daily Tacrolimus in the Early Stage After Living Donor Liver Transplantation. Ther Drug Monit. 2018;40(6):675–81. 10.1097/FTD.0000000000000551 29965882

[30] Lemaitre F, Blanchet B, Latournerie M, Antignac M, Houssel-Debry P, Verdier M-C, et al Pharmacokinetics and pharmacodynamics of tacrolimus in liver transplant recipients: inside the white blood cells. Clinical Biochemistry. Avr 2015;48(6):406–11. 10.1016/j.clinbiochem.2014.12.018 25562187

[31] Tron C, Allard M, Petitcollin A, Ferrand-Sorre M-J, Verdier M-C, Querzerho-Raguideau J, et al Tacrolimus diffusion across the peripheral mononuclear blood cell membrane: impact of drug transporters. Fundam Clin Pharmacol. Févr 2019;33(1):113–21. 10.1111/fcp.12412 30203853

[32] Neely MN, van Guilder MG, Yamada WM, Schumitzky A, Jelliffe RW. Accurate detection of outliers and subpopulations with Pmetrics, a nonparametric and parametric pharmacometric modeling and simulation package for R. Ther Drug Monit. Août 2012;34(4):467–76. 10.1097/FTD.0b013e31825c4ba6 22722776 PMC3394880

[33] Robertsen I, Debord J, Åsberg A, Marquet P, Woillard J-B. A Limited Sampling Strategy to Estimate Exposure of Everolimus in Whole Blood and Peripheral Blood Mononuclear Cells in Renal Transplant Recipients Using Population Pharmacokinetic Modeling and Bayesian Estimators. Clin Pharmacokinet. nov 2018;57(11):1459–69. 10.1007/s40262-018-0646-5 29556934

[34] Bogusz MJ, Enazi EA, Hassan H, Abdel-Jawaad J, Ruwaily JA, Tufail MA. Simultaneous LC-MS-MS determination of cyclosporine A, tacrolimus, and sirolimus in whole blood as well as mycophenolic acid in plasma using common pretreatment procedure. J Chromatogr B Analyt Technol Biomed Life Sci. 1 Mai 2007;850(1‑2):471–80. 10.1016/j.jchromb.2006.12.048 17239667

[35] Blanchet B, Hulin A, Duvoux C, Astier A. Determination of serine/threonine protein phosphatase type 2B (PP2B) in lymphocytes by HPLC. Analytical biochemistry. 2003;312(1):1–6. 10.1016/s0003-2697(02)00214-2 12479828

[36] Pensi D, De Nicolò A, Pinon M, Pisciotta C, Calvo PL, Nonnato A, et al First UHPLC-MS/MS method coupled with automated online SPE for quantification both of tacrolimus and everolimus in peripheral blood mononuclear cells and its application on samples from co-treated pediatric patients. J Mass Spectrom. 2017;52(3):187–95. 10.1002/jms.3909 28098395

[37] Hawwa AF, McElnay JC. Impact of ATP-binding cassette, subfamily B, member 1 pharmacogenetics on tacrolimus-associated nephrotoxicity and dosage requirements in paediatric patients with liver transplant. Expert Opin Drug Saf. janv 2011;10(1):9–22. 10.1517/14740338.2010.505600 20629603

[38] Kravljaca M, Perovic V, Pravica V, Brkovic V, Milinkovic M, Lausevic M, et al The importance of MDR1 gene polymorphisms for tacrolimus dosage. Eur J Pharm Sci. 15 Févr 2016;83:109–13. 10.1016/j.ejps.2015.12.020 26705892

[39] Klaasen RA, Bergan S, Bremer S, Daleq L, Andersen AM, Midtvedt K, et al Longitudinal Study of Tacrolimus in Lymphocytes During the First Year After Kidney Transplantation. Ther Drug Monit. 2018;40(5):558–66. 10.1097/FTD.0000000000000539 30086087

[40] Marquet P, Albano L, Woillard J-B, Rostaing L, Kamar N, Sakarovitch C, et al Comparative clinical trial of the variability factors of the exposure indices used for the drug monitoring of two tacrolimus formulations in kidney transplant recipients. Pharmacol Res. 2018;129:84–94. 10.1016/j.phrs.2017.12.005 29229354

[41] Noceti OM, Woillard J-B, Boumediene A, Esperon P, Taupin J-L, Gerona S, et al Tacrolimus Pharmacodynamics and Pharmacogenetics along the Calcineurin Pathway in Human Lymphocytes. Clinical Chemistry. 1 10 2014;60(10):1336–45. 10.1373/clinchem.2014.223511 25142246

[42] Fukudo M, Yano I, Masuda S, Fukatsu S, Katsura T, Ogura Y, et al Pharmacodynamic analysis of tacrolimus and cyclosporine in living-donor liver transplant patients. Clinical Pharmacology & Therapeutics. Août 2005;78(2):168–81. 16084851 10.1016/j.clpt.2005.04.008

[43] Daher Abdi Z, Prémaud A, Essig M, Alain S, Munteanu E, Garnier F, et al Exposure to mycophenolic acid better predicts immunosuppressive efficacy than exposure to calcineurin inhibitors in renal transplant patients. Clin Pharmacol Ther. 10 2014;96(4):508–15. 10.1038/clpt.2014.140 24968086

