# Supplementary figures and images for: Pharmacogenetic—Whole blood and intracellular pharmacokinetic—Pharmacodynamic (PG-PK2-PD) relationship of tacrolimus in liver transplant recipients
Source: PLoS One. 2020 Mar 12;15(3):e0230195. doi: 10.1371/journal.pone.0230195 (PMC7067455; doi:10.1371/journal.pone.0230195)

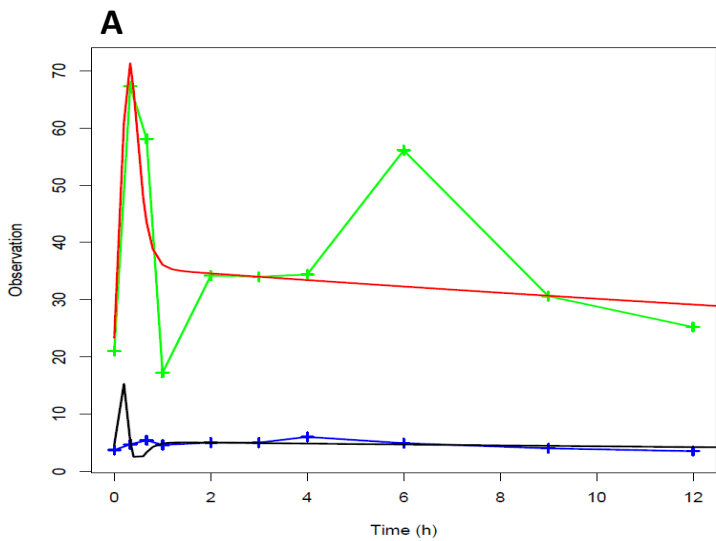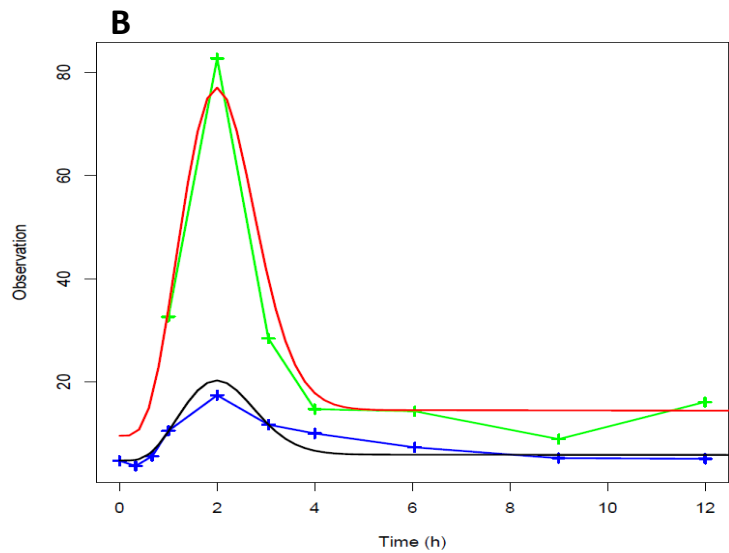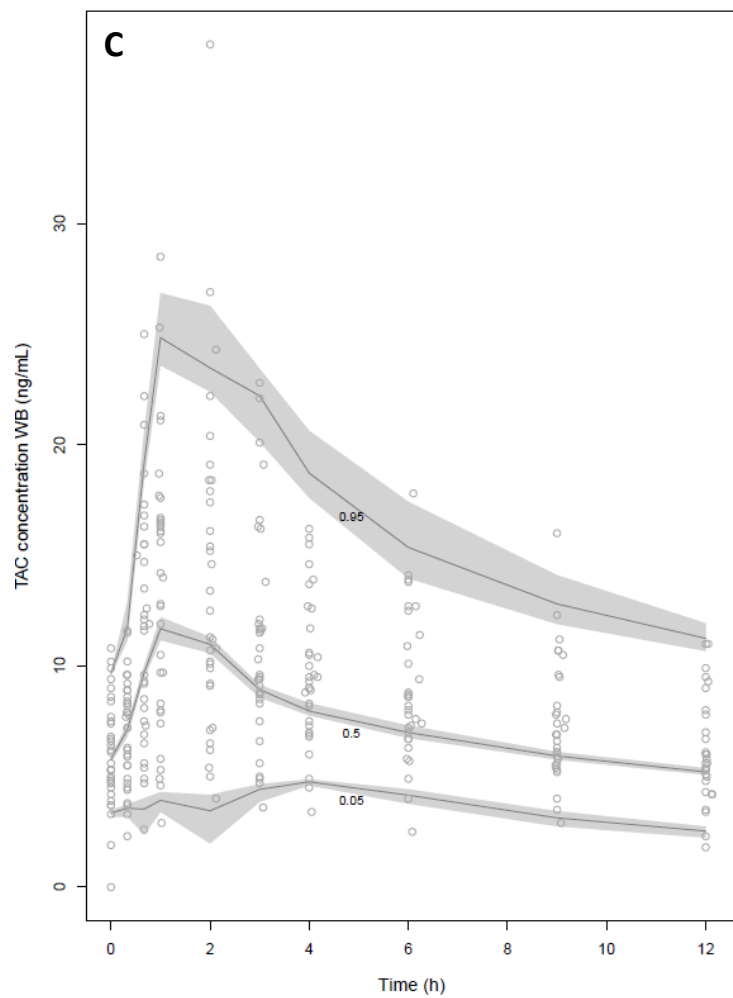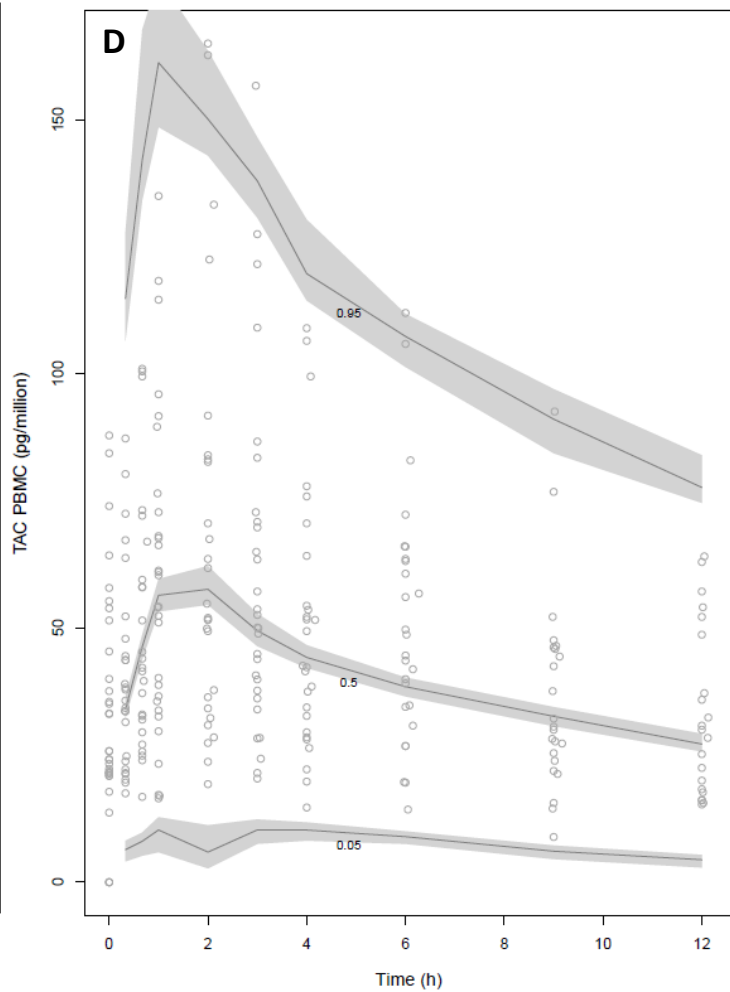

Supplement: S1 Fig — Worst (A) and best (B) individual predicted profiles for tacrolimus (TAC) in whole blood (bottom curve) and in peripheral blood mononuclear cells (PBMC) (upper curve). Green line represents observed tacrolimus concentrations in PBMC, red line represents fitting of the model for PBMC concentrations. Blue line represents observed tacrolimus concentrations in whole blood, black line represents fitting of the model for whole blood concentrations. Visual predictive checks for whole blood (C) and PBMC (D) concentration of tacrolimus. Grey zones are confidence intervals at 95% of 5th, 50th and 95th percentiles of predictions obtained from 1000 Monte-Carlo simulations from the model. Curves are percentiles of the observed data. These curves must be included within the confidence interval above mentioned. (PDF) [file pone.0230195.s001.pdf]

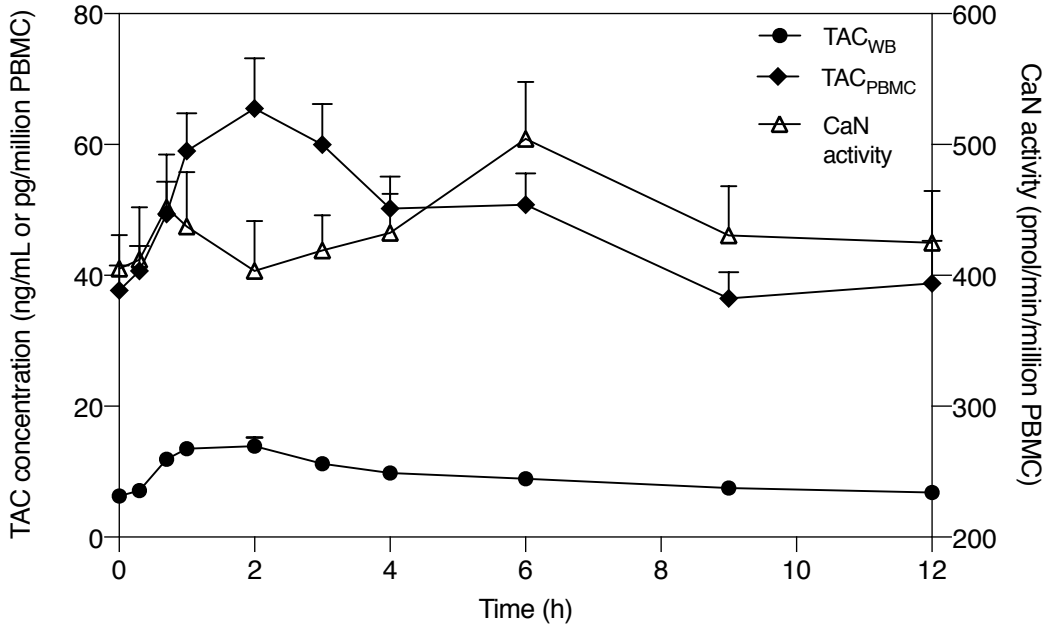

Supplement: S2 Fig — Time course profiles of tacrolimus (TAC) concentrations in whole blood and PBMC (left axis) and calcineurin activity in PBMC (right axis). Each symbol represents mean ± standard deviation of the mean. TACWB: tacrolimus concentration in whole blood, TACPBMC: tacrolimus concentration in PBMC, CaN: calcineurin, PBMC: peripheral blood mononuclear cells. (n = 32). (PDF) [file pone.0230195.s002.pdf]
