# Supplementary material for: Pharmacogenetic—Whole blood and intracellular pharmacokinetic—Pharmacodynamic (PG-PK2-PD) relationship of tacrolimus in liver transplant recipients
Source: PLoS One. 2020 Mar 12;15(3):e0230195. doi: 10.1371/journal.pone.0230195 (PMC7067455; doi:10.1371/journal.pone.0230195)
Supplement: S1 Table — (DOCX) [file pone.0230195.s003.docx]

**Table S1: Population pharmacokinetics parameters**

|  | **Final model parameter estimates** | |  | **Bootstrap value estimates** | |
| --- | --- | --- | --- | --- | --- |
| **Parameter** | **wMedian** | **Interpatient variability (range)** |  | **Median** | **2.5-97.5 percentile** |
| k10(h^-1^) | 0.07 | 0.001-0.22 |  | 0.08 | 0.05-0.09 |
| V1 (L) | 315.3 | 106.9-1498.8 |  | 318 | 248-654 |
| C01 (µg.mL^-1^) | 6.2 | 2.4-10.0 |  | 6.2 | 4.9-7.7 |
| C02 (µg.mL^-1^) | 6.2 | 1.0-33.0 |  | 6.1 | 4.0-13.6 |
| k12 (h^-1^) | 4.7 | 0.4-5.0 |  | 4.7 | 4.4-5.0 |
| k21 (h^-1^) | 4.4 | 0.1-10.0 |  | 4.4 | 3.0-5.7 |
| a1 | 19.9 | 1.0-49.9 |  | 21.8 | 12.6-31.2 |
| b1 (h^-1^) | 7.9 | 0.5-49.9 |  | 7.3 | 6.5-14.0 |
| a2 | 19.9 | 1.0-49.9 |  | 6.2 | 3.5-9.5 |
| b2 (h^-1^) | 0.4 | 0.3-8.1 |  | 0.3 | 0.3-0.9 |
| r | 0.51 | 0.01-0.97 |  | 0.52 | 0.38-0.88 |
| fac | 4.07 | 0.71-49.97 |  | 4.30 | 3.13-6.22 |
| a1, b1, a2, and b2: shape and scale of the two gamma distributions describing the absorption process in the first compartment; k10: elimination rate constant; k12 and k21: rate constant between the compartments; PBMC: peripheral blood mononuclear cells; fac: proportionality factor between the observed PBMC concentration and the concentration estimated by the model [C2obs (t) = fac C2(t)]; r: fraction of dose absorbed in the first compartment following the first gamma function; V1: volume in compartment 1; wMedian: weighted median; C01: model-estimated whole blood trough concentration for a theoretical dose of 1000 mg; C02: model-estimated PBMC trough concentration normalized to 10^6^ cells for a theoretical dose of 1000 mg. | | | | | |
