# Supplementary material for: Pharmacogenetic—Whole blood and intracellular pharmacokinetic—Pharmacodynamic (PG-PK2-PD) relationship of tacrolimus in liver transplant recipients
Source: PLoS One. 2020 Mar 12;15(3):e0230195. doi: 10.1371/journal.pone.0230195 (PMC7067455; doi:10.1371/journal.pone.0230195)
Supplement: S2 Table — (DOCX) [file pone.0230195.s004.docx]

**Table S2: Combined genotypes frequencies**

| **CYP3A4 + CYP3A5 phenotype** | **Recipient phenotype (n)** | | **Donor phenotype (n)** |
| --- | --- | --- | --- |
| intermediate | 30 | | 24 |
| poor | 2 | | 5 |
| extensive | 0 | | 3 |
| **ABCB1 haplotype R/D** | | n | |
| Rhet_TTT/Dhet_TTT | | 7 | |
| Rhet_TTT/Dother | | 7 | |
| Rother/Dhet_TTT | | 5 | |
| Rhet_TTT/Dhom_TTT | | 3 | |
| Rother/Dhom_TTT | | 3 | |
| Rother/Dother | | 3 | |
| Rhom_TTTDhet_TTT | | 2 | |
| Rhom_TTTDother | | 2 | |
| R : recipient, D : donor, het : heterozygote, hom : homozygote, other : other haplotype than TTT for ex 26/12/21 of ABCB1 | | | |
