# Supplementary material for: Pharmacogenetic—Whole blood and intracellular pharmacokinetic—Pharmacodynamic (PG-PK2-PD) relationship of tacrolimus in liver transplant recipients
Source: PLoS One. 2020 Mar 12;15(3):e0230195. doi: 10.1371/journal.pone.0230195 (PMC7067455; doi:10.1371/journal.pone.0230195)
Supplement: S3 Table — (DOCX) [file pone.0230195.s005.docx]

**Table S3: Association analysis between covariates and model parameters**

| **Parameter** | k10(h^-1^) | V1 (L) | C01 (µg.mL^-1^) | C02 (µg.mL^-1^) | k12 (h^-1^) | k21 (h^-1^) | a1 | b1 (h^-1^) | a2 | b2 (h^-1^) | fac | r |
| --- | --- | --- | --- | --- | --- | --- | --- | --- | --- | --- | --- | --- |
| age | 0.25 | 0.87 | 0.23 | 0.98 | 0.41 | 0.23 | 0.57 | 0.26 | 0.71 | 0.11 | 0.27 | *0.004* |
| sex | 0.12 | 0.85 | 0.04 | 0.46 | 0.04 | 0.64 | 0.07 | 0.51 | 0.12 | 0.50 | 0.29 | 0.73 |
| body weight | 0.12 | 0.61 | 0.72 | 0.95 | 0.42 | 0.05 | 0.18 | 0.91 | 0.08 | 0.30 | 0.98 | 0.05 |
| albumin | 0.83 | 0.63 | 0.88 | 0.63 | 0.55 | 0.05 | 0.78 | 0.47 | 0.60 | 0.92 | 0.82 | 0.48 |
| hematocrit | 0.54 | 0.90 | 0.36 | 0.52 | 0.83 | 0.28 | 0.83 | 0.43 | 0.81 | 0.67 | 0.32 | 0.06 |
| count of PBMC | 0.67 | 0.16 | 0.52 | 0.20 | 0.09 | ***0.004*** | 0.11 | 0.81 | 0.76 | 0.07 | 0.72 | 0.39 |
| R ex26 | 0.47 | 0.26 | 0.96 | 0.87 | 0.45 | 0.87 | 0.71 | 0.44 | 0.25 | 0.68 | 0.91 | 0.52 |
| R ex12 | 0.64 | 0.05 | 0.64 | 0.03 | 0.24 | 0.82 | 0.83 | 0.30 | 0.13 | 0.43 | 0.02 | 0.30 |
| R ex 21 | 0.34 | 0.10 | 0.29 | 0.15 | 0.27 | 0.32 | 0.71 | 0.20 | 0.30 | 0.17 | 0.04 | 0.08 |
| R ex 11 | 0.46 | 0.42 | ***0.009*** | 0.61 | 0.10 | 0.92 | 0.65 | 0.37 | 1.00 | 0.29 | 0.44 | 0.48 |
| R haplo | 0.28 | 0.60 | 0.28 | 0.32 | 0.27 | 0.37 | 0.77 | 0.22 | 0.94 | 0.78 | 0.06 | 0.05 |
| R CYP3A4 | 0.13 | 0.26 | 0.13 | 0.41 | 0.41 | 0.13 | 0.16 | 0.61 | 0.91 | 0.81 | 0.70 | 0.97 |
| R CYP3A5 | nc | nc | nc | nc | nc | nc | nc | nc | nc | nc | nc | nc |
| D ex26 | 0.30 | 0.12 | 0.20 | 0.58 | 0.29 | 0.54 | 0.08 | 0.18 | 0.05 | 0.47 | 0.49 | 0.19 |
| D ex12 | 0.50 | 0.87 | 0.84 | 0.31 | 0.42 | 0.62 | 0.01 | 0.39 | 0.45 | 0.85 | 0.43 | 0.56 |
| D ex 21 | 0.16 | 0.63 | 0.33 | 0.23 | 0.91 | 0.56 | 0.00 | 0.97 | 0.30 | 0.54 | 0.60 | 0.19 |
| D ex 11 | 0.52 | 0.45 | 0.10 | 0.16 | 0.16 | 0.10 | 0.59 | 0.19 | 0.94 | 0.29 | 0.39 | 0.10 |
| D haplo | 0.74 | 0.59 | 0.57 | 0.97 | 0.51 | 0.91 | *0.01* | 0.05 | 0.89 | 0.84 | 0.61 | 0.74 |
| D CYP 3A4 | 0.80 | 0.29 | 0.53 | 0.72 | 0.36 | 0.41 | 0.60 | 0.45 | 0.84 | 0.67 | 0.57 | 0.55 |
| D CYP 3A5 | 0.61 | 0.92 | 0.27 | 0.35 | 0.26 | 0.48 | 0.58 | 0.42 | 0.25 | 0.27 | 0.50 | 0.52 |
| Phenotype of metabolisation R | 0.12 | 0.24 | 0.12 | 0.39 | 0.39 | 0.12 | 0.15 | 0.59 | 0.88 | 0.78 | 0.67 | 0.94 |
| Phenotype of metabolisation D | 0.71 | 0.54 | 0.26 | 0.57 | 0.64 | 0.38 | 0.54 | 0.26 | 0.53 | 0.55 | 0.83 | 0.50 |
| ABCB1_R and D | 0.79 | 0.95 | 0.85 | 0.72 | 0.76 | 0.71 | 0.06 | 0.11 | 0.97 | 0.73 | 0.44 | 0.39 |
| Data are p-value of statistical test were reported in the table. Interaction between covariates and model parameters were analyzed using Kruskal-Wallis (+ post hoc analysis with Bonferroni correction) or Mann-Whitney for categorical covariates and linear regression for continuous covariates. If significant association was found with p<0.01, covariates were individually introduced in the model to check whether it could improve the prediction (AIC). | | | | | | | | | | | | |
| nc: not calculable, D: donor, R: recipient | | | | | | | | | | | | |
