# Supplementary material for: Pharmacogenetic—Whole blood and intracellular pharmacokinetic—Pharmacodynamic (PG-PK2-PD) relationship of tacrolimus in liver transplant recipients
Source: PLoS One. 2020 Mar 12;15(3):e0230195. doi: 10.1371/journal.pone.0230195 (PMC7067455; doi:10.1371/journal.pone.0230195)
Supplement: S4 Table — (DOCX) [file pone.0230195.s006.docx]

**Table S4: Hardy-Weinberg equilibrium analysis**

| **Genotype** | **Allelic status** | **n** | p-value |
| --- | --- | --- | --- |
| Recipient ABCB1 3435C>T | **CC** | 2 | 0.03 |
| (rs1045642) | **CT** | 21 |  |
|  | **TT** | 9 |  |
| Recipient ABCB1 1236 C>T | **CC** | 10 | 0.76 |
| (rs1128503) | **CT** | 15 |  |
|  | **TT** | 7 |  |
| Recipient ABCB1 2677 G>T/A | **GG** | 11 | 0.83 |
| (rs2032582) | **GT** | 15 |  |
|  | **TT** | 6 |  |
| Recipient ABCB1 1199 G>A | **GG** | 29 | 0.78 |
| (rs2229109) | **GA** | 3 |  |
| Recipient CYP3A4 | **CC** | 30 | 0.86 |
| (C>T) |  |  |  |
| (*22, rs35599367) | **CT** | 2 |  |
| Recipient CYP3A5 | **AA** | 32 |  |
| 6986 G>A |  |  | / |
| (*3, rs776746) |  |  |  |
| Donor ABCB1 3435C>T | **CC** | 8 | 0.18 |
| (rs1045642) | **CT** | 12 |  |
|  | **TT** | 12 |  |
| Donor ABCB1 | **CC** | 11 | 0.31 |
| 1236 C>T | **CT** | 13 |  |
| (rs1128503) | **TT** | 8 |  |
| Donor ABCB1 | **GG** | 10 | 0.08 |
| 2677 G>T/A | **GT/A** | 11 |  |
| (rs2032582) | **TT/A** | 11 |  |
| Donor ABCB1 | **GG** | 31 | 0.93 |
| 1199 G>A |  |  |  |
| (rs2229109) | **GA** | 1 |  |
| Donor CYP3A4 | **CC** | 27 | 0.63 |
| (C>T) |  |  |  |
| (*22, rs35599367) | **CT** | 5 |  |
| Donor CYP3A5 | **Expressor** | 1/2 | 0.01 |
| 6986 G>A | **GG and GA** |  |  |
| (*3, rs776746) | **Non expressor** | 29 |  |
|  | **AA** |  |  |
